# Decreased serpin C1 in extracellular vesicles predicts response to methotrexate treatment in patients with pulmonary sarcoidosis

**DOI:** 10.1186/s12931-024-02809-y

**Published:** 2024-04-16

**Authors:** Raisa Kraaijvanger, Montse Janssen Bonás, Jan C. Grutters, Ioanna Paspali, Marcel Veltkamp, Dominique P. V. de Kleijn, Coline H. M. van Moorsel

**Affiliations:** 1 Department of Pulmonology, St Antonius Hospital, Interstitial Lung Diseases Center of Excellence, Nieuwegein, The Netherlands; 2 Division of Heart and Lungs, University Medical Center, Utrecht, The Netherlands; 3 Department of Vascular Surgery, University Medical Center, Utrecht, The Netherlands

**Keywords:** Extracellular vesicles, Sarcoidosis, Biomarkers, Therapy

## Abstract

**Background:**

Sarcoidosis is a systemic granulomatous disease of unknown etiology primarily affecting the lungs. Treatment is needed when disease symptoms worsen and organ function deteriorates. In pulmonary sarcoidosis, prednisone and methotrexate (MTX) are the most common anti-inflammatory therapies. However, there is large inter-patient variability in response to treatment, and predictive response markers are currently lacking.

**Objective:**

In this study, we investigated the predictive potential of biomarkers in extracellular vesicles (EVs) isolated from biobanked serum of patients with pulmonary sarcoidosis stored prior to start of therapy.

**Methods:**

Protein concentrations of a four-protein test panel of inflammatory proteins were measured in a discovery (*n* = 16) and replication (*n* = 129) cohort of patients with sarcoidosis and 47 healthy controls. Response to therapy was defined as an improvement of the absolute score of > 5% forced vital capacity (FVC) and/or > 10% diffusion lung of carbon monoxide (DLCO) after 24 weeks compared to baseline (before treatment).

**Results:**

Serum protein levels differed between EV fractions and serum, and between sarcoidosis cases and controls. Serpin C1 concentrations in the low density lipid particle EV fraction were lower at baseline in the group of patients with a good response to MTX treatment in both the discovery cohort (*p* = 0.059) and in the replication cohort (*p* = 0.032). EV Serpin C1 showed to be a significant predictor for response to treatment with MTX (OR 0.4; *p* = 0.032).

**Conclusion:**

This study shows that proteins isolated from EVs harbor a distinct signal and have potential as new predictive therapy response biomarkers in sarcoidosis.

**Supplementary Information:**

The online version contains supplementary material available at 10.1186/s12931-024-02809-y.

## Introduction

Sarcoidosis is a systemic granulomatous disease of unknown cause mainly affecting the lungs, intrathoracic lymph nodes, eyes and skin [1]. Diagnosis, monitoring, as well as predicting disease course or response to therapy is challenging in the management of patients with sarcoidosis. For decades, biomarkers such as angiotensin converting enzyme (ACE) and soluble interleukin-2 receptor (sIL-2R) have been studied to guide clinical management, unfortunately with modest sensitivity and specificity [2, 3]. Pharmacological treatment of sarcoidosis is initiated to prevent further specific organ damage or alleviate symptoms. Immunosuppressing and immunomodulating drugs, most often in the form of prednisone and methotrexate (MTX) are the first- and second-line choice of therapy. When initiating therapy however, it is not possible to predict the treatment response for individual patients upfront, while a significant part show no benefit from therapy. Personalized prediction of treatment response is a clinical unmet need in light of protecting patients from exposure to ineffective drugs and their side effects.

Sarcoidosis is characterized by the formation of non-caseating granulomas, persistent inflammation and activated monocytes/macrophages [4]. Macrophages form the core of the granuloma, producing inflammatory cytokines and chemokines to attract lymphocytes resulting in an inflammatory environment [5]. These macrophages originate from circulating bone-marrow derived monocytes which are patrolling antigen presenting cells but have a secondary function as a reservoir to replenish the macrophage pool in the tissues when needed. Several studies have highlighted an increased inflammatory status of monocytes in the blood of sarcoidosis patients [6–9]. This increased inflammatory status can also be induced through extracellular vesicles (EVs) as has been described previously by Wahlund et al. [9]. Furthermore, more monocytes are found in the circulation of patients with sarcoidosis and these cells have the capacity to activate other immune cells, not only through cell-cell interaction but also through monocyte derived EVs [10, 11]. Taken together, EVs isolated from whole blood serum may be particularly informative in sarcoidosis.

EV is an umbrella term for all vesicles found in body fluids, including exosomes, micro-vesicles and apoptotic bodies. In the last decade, it was found that EVs can functionally transfer molecules between cells [12]. EVs contain nucleic acids, lipids and proteins from the releasing cells and are often referred to as liquid biopsies [13, 14] reflecting the status/pathology of the releasing cell. EV proteins are often better associated with the pathology then the same freely circulating proteins in blood [15]. In the search for new potential biomarkers, there has been an increased interest in EVs [16–18]. Because of the relatively new stage of this emerging field of research there is a need for standardization in both methodology and technology to be able to validate the EV-associated biomarkers [19].

For this study a well-established EV-protein panel [20] was used, originally developed to predict adverse cardiovascular events. This panel consisted of four proteins, CD14, Serpin G1, Serpin C1 and Cysteine C. Although, the four proteins in this panel are mostly used in the field of cardiology, these proteins are also involved in inflammation and coagulation [21–27]. Therefore, this panel of proteins could be of interest in assessment of inflammation and prediction of response to immunosuppressive treatment in sarcoidosis. The proteins were originally measured in form of a multiplex, however, for this study the proteins were measured separately to investigate the associations of the individual proteins to response to treatment.

In this exploratory study, we investigated the potential of inflammatory biomarkers derived from serum-isolated fractions of EV using a well-established panel of proteins previously validated in EVs. The goal of the present study was three-fold. First, we investigated whether differences in levels of inflammatory biomarkers exist between EV-isolates and serum. Second, we investigated whether EV-derived biomarkers differ between patients with sarcoidosis and healthy controls. Third, we assessed whether these inflammatory biomarkers predict treatment response in patients using prednisone or MTX.

## Materials and methods

### Patients

Case and control samples were collected from the St. Antonius Hospital ILD biobank, screening all sarcoidosis patients (*n* = 2265) for eligibility. Cases were selected based on pulmonary treatment indication (decrease in lung function, dyspnea or pulmonary fibrogenesis), treatment with prednisone or MTX, and presence of serum collected within 6 months prior to start of treatment. 98 sarcoidosis patients were treated with MTX and 69 patients treated with prednisone. All patients were adults > 18 years with sarcoidosis diagnosed according to the international ATS/ERS/WASOG criteria [1].

Baseline characteristics (age at diagnosis, gender, co-morbidities as reported in the medical records), lung function, Löfgren syndrome as well as organ manifestation were recorded up to 2 years after start of treatment. Based on the results of the recently reported SARCORT trial [28] as well as the sarcoidosis treatment score [29], an improvement of the absolute score of > 5% forced vital capacity (FVC) and/or > 10% diffusion lung of carbon monoxide (DLCO) after 24 weeks compared to baseline (before treatment) classified a patient as a “responder”. All other patients were classified as “non-responder”.

This study was performed in accordance with the Declaration of Helsinki and GCP guidelines. The study was approved by the Medical research Ethics Committees United (MEC-U) of the St. Antonius Hospital (R05-08A) and written consent was obtained from all patients.

### Study cohorts

**Fig. 1:**
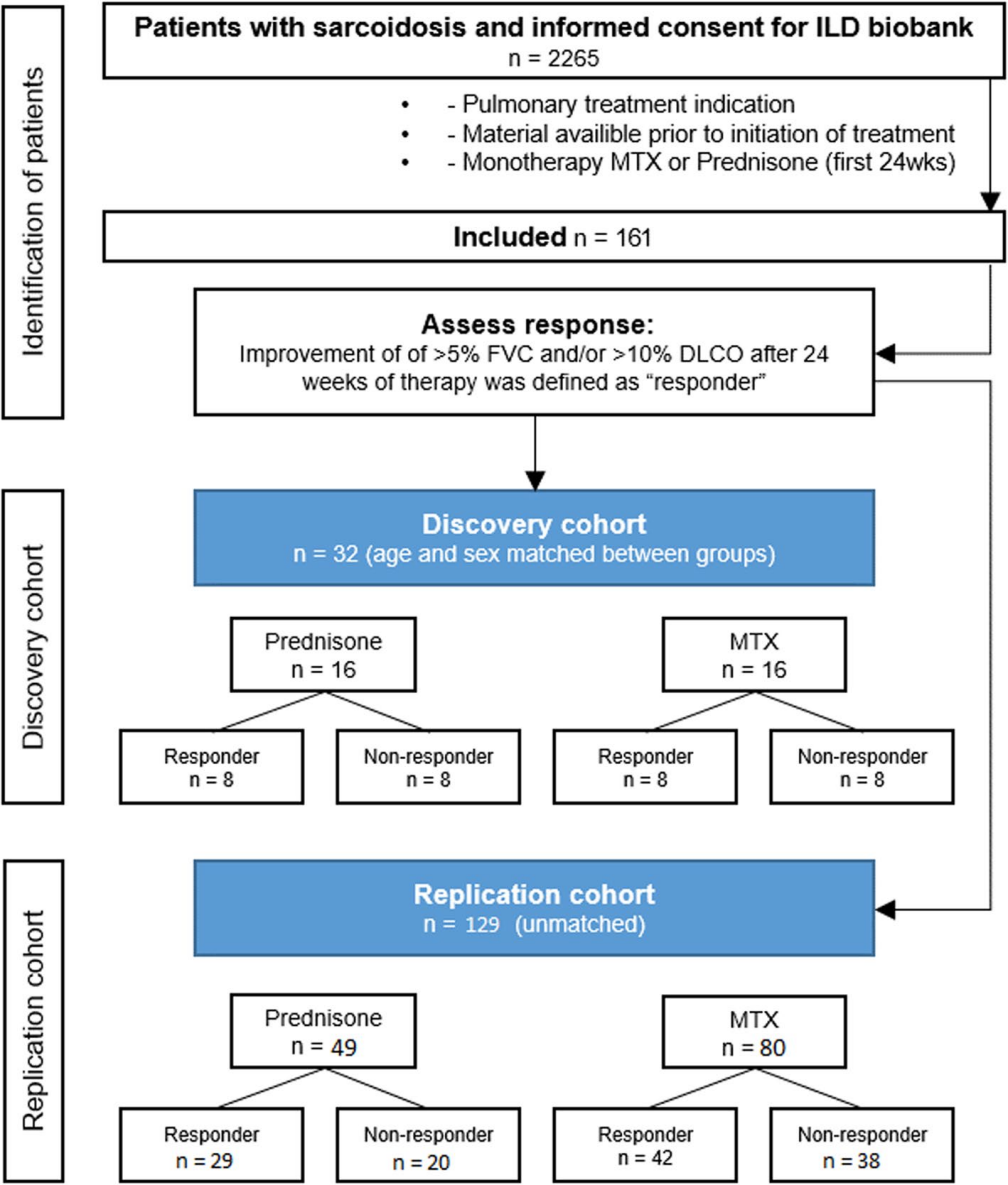
Flowchart showing procedure for identification of patients and distribution of patients across the discovery and replication cohort. In brief, from 2265 patients with sarcoidosis and informed consent for ILD biobank 161 patients fulfilled the criteria to be included in the study. A total of 32 sex and age matched patients were selected for the discovery cohort. The remaining 129 patients were included in the replication cohort

First the discovery cohort was composed, a cohort of 16 prednisone and 16 MTX cases which were matched on age, sex, ethnicity and smoking history as best as possible. For each treatment, eight patients were responders and eight patients were non-responders to therapy. The replication cohort consisted of the remainder 49 prednisone and 80 MTX cases. In the replication cohort the prednisone treated group consisted of 29 responders and 20 non-responders, and the MTX treated group consisted of 42 responders and 38 non-responders. In addition, 47 healthy control samples were included. The workflow is summarized in a flowchart in Fig 1.

Blood samples were collected before any treatment was given. Serum was isolated by the centrifugation of serum separator clot activator tubes at 1800 g for 5 minutes and stored at − 80 °C in the St. Antonius Hospital BIOBANK until further use.

### EV isolation

EV fraction isolation was based on the protocol of Dekker et al. [30]; however, here serum instead of plasma was used (see detailed description in the supplemental materials). EVs were isolated from 25 μL serum with the use of magnetic beads (Nanomag®-D plain, 130 mm (1:25) (Micromod). For sequential isolation of the fractions Dextran Sulphate (DS) (MP Biomedicals, Illkrich, France) and Manganese (II) chloride (MnCl_2_) solution (Sigma Aldrich, St. Louis, MO, USA) were used. The presence of isolated EVs using this technique was previously confirmed with electron microscopy and western blotting [30–32]. Different fractions of EVs in serum were obtained by EV co-precipitation with monolayer low-density lipid particles (LDL fraction) and with bilayer membrane vesicles high-density lipid particles (HDL fraction). Previous performed experiments have shown that relatively small EVs (± 101 nm) are present in the LDL fraction while larger particles are found in the HDL fraction (± 120 nm) [30].

### Protein concentration measurements

On a 96-wells plate, LDL, HDL and whole serum were measured simultaneously. Protein concentrations in EV fractions and serum were measured in a well-established protein panel consisting of: CD14, cystatin C, serpin C1 and serpin G1 [20]. Capture antibody, biotinylated detection antibody and antigen of all four proteins were purchased from R&D systems. Proteins were quantitatively analyzed by Luminex-based multiplex assay (Bio-Rad, Austin, USA). All EV protein levels were corrected for total amount of protein.

### Statistical analysis

Non-parametric Mann-Whitney U test was used for non-normally distributed data. Categorical variables were compared using Chi-Squared and Fisher’s exact test, where appropriate. Log transformed values of the protein levels were used to reduce the effect of skewness in the distribution of the protein-levels. To calculate the odds ratio and enable the direct comparison between different proteins, EV-protein levels were converted into standardized units, or the z-score, by using the observed value minus the mean value, divided by the standard deviation. To investigate the relationship between the protein levels and response to therapy, a logistic regression model was used with the outcome “response to medication”. Spearman’s rho correlations were calculated to assess direct relationships between protein concentrations and other parameters. For the analysis in the discovery cohort *p*-values < 0.1 were considered of significant interest, for the analysis with the replication cohort and the combined cohort *p*-values < 0.05 were considered significant.

## Results

### Discovery cohort

#### Baseline characteristics

Baseline characteristics of the age, sex, ethnicity and smoking history matched discovery cohort of the patient groups treated with MTX or prednisone are shown in Table 1. For the MTX group the DLCO %pred was significantly lower in the responding group (*p* = 0.010).

**Table 1:** Baseline characteristics of discovery cohort of sarcoidosis patients with pulmonary treatment indication

Parameter		Prednisone treated group		Methotrexate treated group	
		Non-responding (*n* = 8)	Responding (*n* = 8)	Non-responding (*n* = 8)	Responding (*n* = 8)
**Age**^**a**^ **(years)**		43.3 ± 6.7	37.4 ± 8.5	48.3 ± 7.0	52.6 ± 13.0
**Male sex**		5 (62.5)	5 (62.5)	5 (62.5)	5 (62.5)
**Ever smoker**		2 (25.0)	2 (25.0)	2 (25.0)	2 (25.0)
**Caucasian**		8 (100)	8 (100)	8 (100)	8 (100)
**Lofgren syndrome**		0 (0.0)	1 (12.5)	1 (12.5)	0 (0.0)
**Scadding stage**^**b**^ **0/I/II/III/IV**		1/2/2/1/2 (12.5/25/25/12.5/25)	1/1/3/1/2 (12.5/12.5/37.5/12.5/25)	0/1/5/0/2 (0/12.5/62.5/0/25)	0/1/4/1/2 (0/12.5/50/12.5/25)
**Lung function**^**b**^					
	FVC (%)	94.5 ± 28.5	81.8 ± 24.2	102.7 ± 11.6	89.0 ± 21.8
	DLCO (%)	75.8 ± 25.0	66.1 ± 13.2	86.1 ± 12.6*	55.5 ± 13.1*
**Extra-pulmonary involvement**					
	Lymph nodes	5 (62.5)	4 (50.0)	5 (62.5)	4 (50.0)
	Skin	0 (0.0)	1 (12.5)	0 (0.0)	1 (12.5)
	Liver	0 (0.0)	0 (0.0)	1 (12.5)	0 (0.0)
	Spleen	1 (12.5)	1 (12.5)	1 (12.5)	0 (0.0)
	SFN	0 (0.0)	0 (0.0)	0 (0.0)	0 (0.0)

Data is shown as whole numbers and percentages between brackets. Response to treatment was based on improvement in lung function (FVC %pred > 10% or DLCO %pred > 10%) after 6 months of treatment. Age, lung function, and biomarkers are shown as mean ± SD. ^a^Age at time of blood withdrawal. ^b^lung function and scadding stage were measured before start of treatment. Scadding stages: 0 = Normal chest radiograph; I = Bilateral hilar lymphadenopathy (BHL); II = BHL with pulmonary infiltrates; III = pulmonary infiltrates without BHL; IV = fibrosis. SFN: Small fiber neuropathy. DLCO (%) was significantly lower in patients responding to MTX therapy. *p < 0.05

#### Differences in protein levels between EV and serum in sarcoidosis

**Fig. 2:**
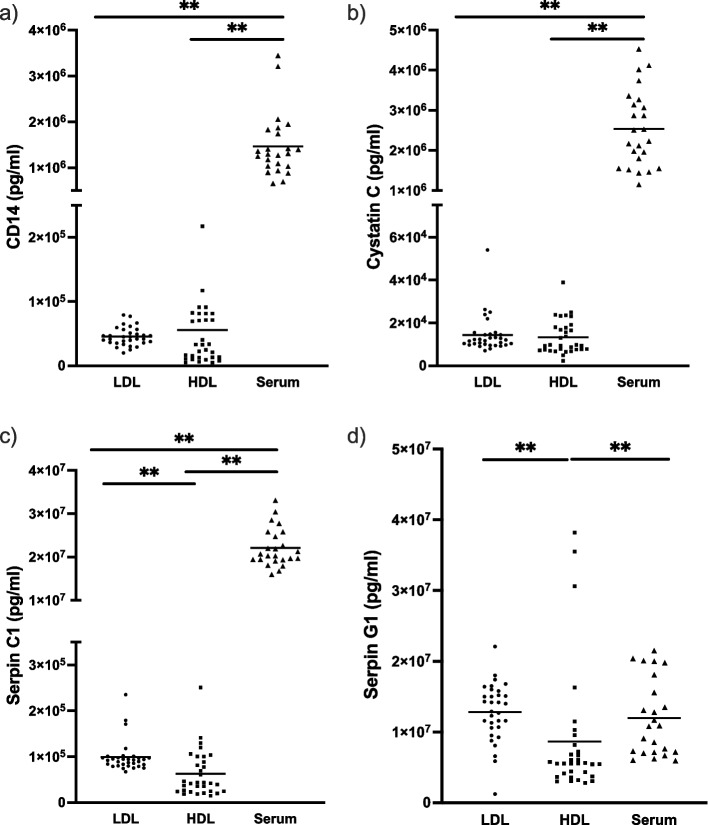
Concentrations of proteins measured in different EV fractions (LDL and HDL) and in whole serum in the sarcoidosis discovery cohort (*n* = 32). For all proteins except serpin G1 serum concentrations were higher compared to EV-fractions (LDL and HDL). For both serpin G1 and C1 there was a difference between concentrations in LDL and HDL. LDL = low-density lipid, HDL = high-density lipid. **p < 0.005

The proteins from the protein test panel were measured in the EV-fractions LDL, HDL and in whole serum. Figure 2 shows the concentrations of the different proteins measured in the discovery cohort (*n* = 32). Compared with EV-fractions, whole serum protein concentrations were higher for CD14, Cystatin C and Serpin C1, but not for Serpin G1. Furthermore, protein concentrations for Serpin G1 and Serpin C1 were lower in the HDL fraction than in the LDL faction.

#### EV-derived proteins are higher in sarcoidosis than in controls

**Fig. 3:**
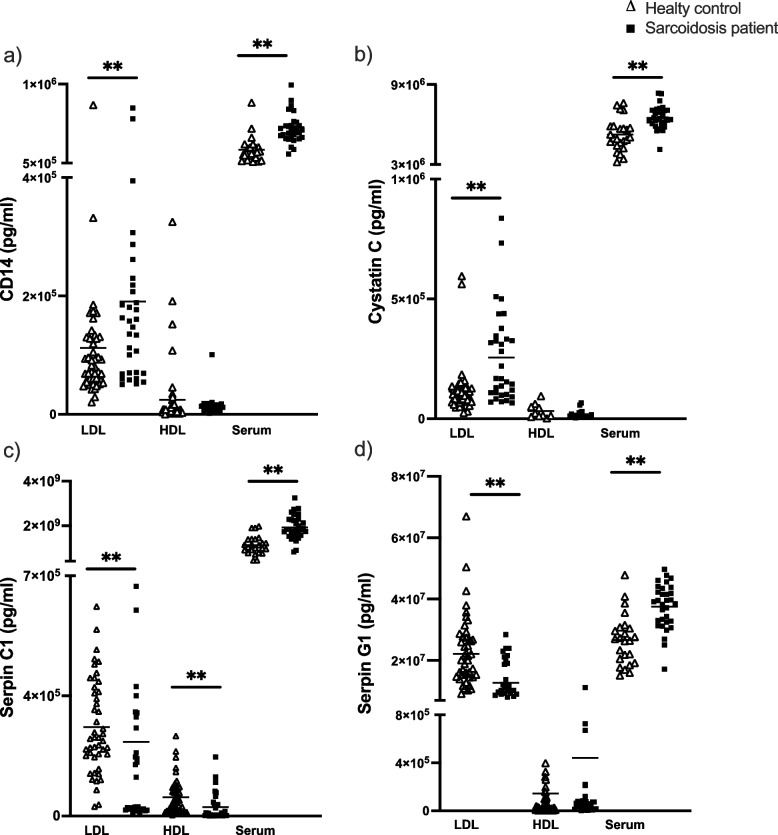
Concentrations of proteins from EV-protein test panel. Light triangles represent healthy controls and dark squares represent sarcoidosis patients from the discovery cohort. **a** Concentrations of CD14; (**b**) concentrations of cystatin C; (**c**) concentrations of serpin C1; and (**d**) concentrations of serpin G1 measured in EV fractions LDL and HDL and in whole serum. LDL = low-density lipid, HDL = high-density lipid. **p < 0.005

The proteins from the protein test panel measured in the discovery cohort were compared to protein levels in healthy controls (HC) (*n* = 47). Baseline characteristics are shown in Supplementary Table S1. The concentration of proteins was significantly higher in sarcoidosis patients compared to HC when measured in whole serum (Fig. 3). For the HDL EV-fraction only serpin C1 was significantly different between patients and HC. For the LDL fraction, a significant difference was observed for all proteins of the test panel. When compared to healthy controls, the concentrations of CD14 and cystatin C were higher in the LDL fractions of patients while concentrations of both serpin proteins were lower in LDL fractions.

#### Response to treatment

In the prednisone discovery cohort no differences were found in protein concentrations between responders and non-responders in either LDL, HDL fractions or serum.

**Fig. 4:**
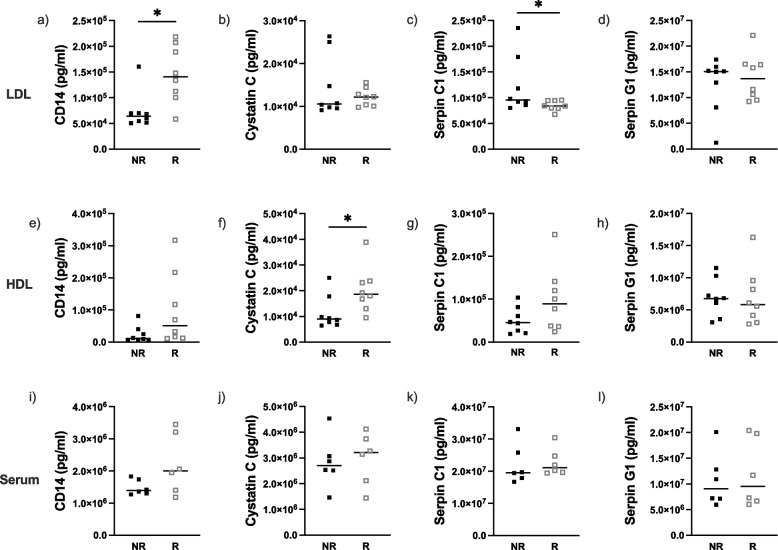
Concentrations of proteins from EV-protein test panel in patients with sarcoidosis treated with methotrexate of the discovery cohort (*n* = 16). Figures (**a**-**d**) represents EV-proteins measured in the LDL fraction, figures (**e**-**h**) EV-proteins measured in the HDL fraction and figures (**i**-**l**) proteins measured in whole serum. Filled squares represent non-responders (NR) and open squares represent responders (R) to treatment with methotrexate. *p < 0.1

In the MTX discovery cohort, concentrations of serpin C1 were lower (*p* = 0.059) in the group of responders while concentrations of CD14 were significantly higher in the group of patients classified as responders (*p* = 0.014) in the LDL fraction. In the HDL fraction, cystatin C concentrations were lower in the group of patients classified as non-responders (*p* = 0.027) (Fig. 4). No difference was found in serum protein levels of responders and non-responders (Supplementary Fig. S1).

### Replication cohort

#### Baseline characteristics

In the second phase of the study, the EV proteins were quantified in a larger replication cohort of 80 sarcoidosis patients treated with methotrexate and 49 sarcoidosis patients treated with prednisone. Baseline characteristics are shown in Table 2. As with the discovery cohort, all included patients had a pulmonary treatment indication.

**Table 2:** Baseline characteristics of the replication cohort of sarcoidosis patients with pulmonary treatment indication

Parameter		Prednisone treated group		Methotrexate treated group	
		Non-responding (*n* = 20)	Responding (*n* = 29)	Non-responding (*n* = 38)	Responding (*n* = 42)
**Age**^**a**^ **(years)**		43.6 ± 10.0	43.5 ± 9.6	49.0 ± 10.0	47.9 ± 10.9
**Male sex**		14 (70.0)	21 (72.4)	23 (71.9)	25 (59.5)
**Ever smoker**		5 (31.3)	10 (47.6)	15 (60.0)	18 (52.9)
**Caucasian**		18 (90.0)	26 (92.9)	28 (90.3)	36 (90.0)
**Lofgren syndrome**		0 (0.0)	2 (7.1)	2 (6.3)	2 (4.8)
**Scadding stage**^**b**^ **0/I/II/III/IV**		0/2/12/1/4 (0/11/63/5/21)	0/5/14/1/7 (0/19/52/4/26)	2/3/11/1/12 (7/10/38/3/4130)	2/7/18/0/7 (6/20/53/0/21)
**Lung function**^**b**^					
	FVC (%)	94.5 ± 19.1	83.5 ± 20.4	97.1 ± 22.2	91.1 ± 23.1
	DLCO (%)	70.3 ± 16.3	66.6 ± 17.8	72.3 ± 15.0	67.7 ± 15.5
**Extra-pulmonary involvement**					
	Lymph nodes	12 (60.0)	16 (55.2)	22 (68.8)	28 (66.7)
	Skin	0 (0.0)	3 (10.3)	6 (18.8)	4 (9.5)
	Liver	1 (5.0)	1 (3.4)	5 (15.6)	6 (14.3)
	Spleen	5 (25.0)	5 (17.2)	7 (21.9)	7 (16.7)
	SFN	4 (20.0)	2 (6.9)	5 (15.6)	3 (7.1)

Data is shown as whole numbers and percentages between brackets. Response to treatment was based on improvement in lung function (FVC %pred > 10% or DLCO %pred > 10%) after 6 months of treatment. Age, lung function, and biomarkers are shown as mean ± SD. ^a^Age at time of blood withdrawal. ^b^lung function and scadding stage were measured before start of treatment. Scadding stages: 0 = Normal chest radiograph; I = Bilateral hilar lymphadenopathy (BHL); II = BHL with pulmonary infiltrates; III = pulmonary infiltrates without BHL; IV = fibrosis. SFN: Small fiber neuropathy

**Fig. 5:**
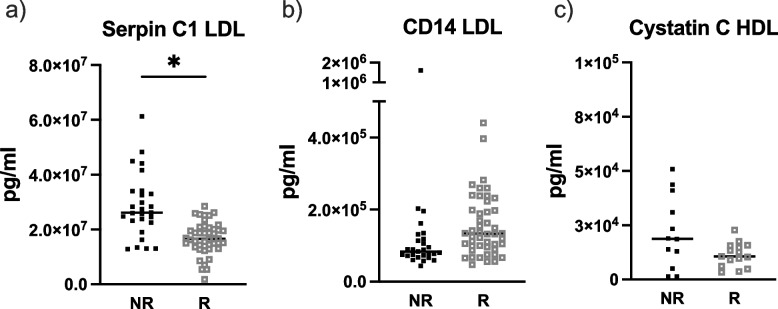
Concentrations of EV proteins in patients with sarcoidosis treated with methotrexate of the replication cohort (*n* = 82), measured in different EV fractions as well as in whole serum. Figure (**a**) represents concentrations of Serpin C1 in the LDL fraction; figure (**b**) concentrations of CD14 in the LDL fraction, and figure (**c**) concentrations of cystatin C in the HDL fraction in non-responders and responders to treatment with methotrexate. Serpin C1 concentrations were significantly higher in non-responders than in responders (*p* = 0.032). Black filled squares represent non-responders (NR) and gray open squares represent responders (R) to treatment with methotrexate. *p < 0.05

Supplementary Fig. S2 shows the concentrations of all proteins measured in the replication cohort. Regarding the proteins of interest identified in the discovery cohort, serpin C1 concentrations in the LDL fraction were significantly lower in the methotrexate responder than in the non-responder group of the replication cohort (*p* = 0.032; Fig. 5a). Regarding CD14 in LDL and cystatin C in HDL, however, no difference between responders and non-responders was found (*p* = 0.091 and *p* = 0.215, respectively, Fig. 5b, c).

#### EV proteins to predict response to MTX

To determine the predictive value of serpin C1 for treatment response prediction we combined the MTX discovery and replication cohorts for further analysis. Values were log-transformed to stabilize the variance. Logistic regression revealed that serpin C1, when measured in the LDL sub fraction, was a significant predictor for response to treatment (OR 0.42 [95%CI 0.19–0.93] *p* = 0.032).

To investigate if there was a direct relation, Spearman’s rank correlations were computed to assess the relation between change in FVC 6 months after initiation of MTX therapy and protein concentrations at baseline. There was a positive correlation between change in FVC and CD14 concentrations in the LDL fraction, (R = 0.35; *p* = 0.004). None of the other proteins directly correlated with change in FVC and no correlation was found for change in DLCO.

## Discussion

In this study, we showed that concentrations of proteins in serum and in EVs fractions isolated from serum can differ significantly from each other and that this depends on the analyzed protein. Second, we found that levels of proteins in EV fractions differed significantly between sarcoidosis and healthy controls. Third, we showed that EV proteins, and particularly serpin C1 in the LDL EV-fraction is significantly lower at baseline in the group of patients that responded to treatment with MTX, which we verified in a second cohort. Baseline serpin C1 was shown to be a marker with predictive value of response to MXT therapy.

Our data demonstrates that measuring proteins specifically in EV can provide novel information in sarcoidosis research compared to measuring the same proteins in serum alone. Although EVs have the disadvantage of an extra isolation step, they do also have advantages such as stability, easy accessibility and minimal sample volumes [18]. Furthermore, EVs are increasingly recognized to contain proteins involved in cellular processes directly linked to disease pathogenesis [33].

The difference in protein expression between patients and healthy controls is more evident in the LDL fraction than in the HDL fraction. A possible explanation for this observation lies in the difference in composition of the vesicles, HDL vesicles are larger and are suggested to contain more proteins from the originating cells [34, 35]. While the LDL fraction consist of smaller EVs including exosomes which are released by activated immune cells and have the capacity to activate other immune cells. The overly active immune system in sarcoidosis patients could lead to an increased release in exosomes eventually resulting in higher EV protein concentrations in patients versus healthy controls [36]. Protein concentrations of pro-inflammatory cytokines CD14 and Cystatin C have been shown to be elevated in multiple inflammatory disorders [37, 38] and were also elevated in the LDL fraction in sarcoidosis patients compared to healthy controls. Both serpin C1 and G1 were elevated in healthy controls. These serpins exert anti-inflammatory properties [21, 39]. However, as previous research describes a decrease in EV numbers with advancing age [40]. We also have to take into account the possibility of an age effect on the differences in EV-protein concentrations between patients and healthy controls, since the healthy controls were significantly younger. When comparing the concentrations of the proteins with the age of the healthy control subject, none of the proteins correlated with age resulting in a minimal impact of age on the difference in EV-protein concentrations between patients and healthy controls.

In light of diagnostic potential of these biomarkers, there was no added value of EV over serum since all four proteins also differed significantly between patients and healthy controls in serum. Further analysis, using serum samples from time of diagnosis from patients with sarcoidosis and differential diagnoses are needed to determine the diagnostic potential of the markers. The EV proteins in our panel are general inflammation markers, found on the cell surface, shed from the cells or actively involved in cellular inflammatory processes. Therefore, it is not surprising that we found a difference in protein concentrations between healthy controls and patients. However, an interesting finding was made for serpin C1. In the LDL and HDL EV-fractions, serpin C1 concentrations were lower in patients than in controls, while in whole serum, the serpin C1 concentration was higher in patients than in controls. The effect of serpin C1 is different in the circulation than as a cellular surface maker, where it has an increased anti-inflammatory effect. As previously suggested, more serpin C1 may have been shed in patients with sarcoidosis compared to healthy controls [41], which corresponds to our increased levels in the serum of patients.

Further aim of investigation was to assess whether protein concentrations in EV could have a predictive value for response to therapy. To assess this, patients receiving either prednisone or MTX were divided in responders and non-responders, based on change in lung function. The protein test panel revealed no difference in EV-fractions between responders and non-responders in the group of patients treated with prednisone. For the patient group treated with MTX, Serpin C1 concentrations were significantly lower in the group of patients responding to treatment while CD14 concentrations were higher in the responder group, unfortunately for CD14 the difference between responders and non-responder was no longer significant. In addition, we found a positive correlation between CD14 concentrations at baseline and change in FVC over the course of 6 months of MTX treatment (*p* = 0.004). This protein was previously identified as an EV marker for sarcoidosis by Futami et al., who described CD14 levels in EVs to be significantly increased in patients with sarcoidosis and showed that CD14 was up-regulated in the process of granuloma formation [42]. In MTX treated patients with RA a positive correlation has been described between the concentration of soluble CD14 (sCD14) and response to MTX, RA patients with the highest sCD14 concentration at baseline responded best to MTX treatment. Our findings suggest a similar effect as responders have the highest concentration of CD14 in the vesicles at baseline and this correlates with highest change in FVC [43]. In future, it would be interesting to see how the CD14 concentration in EVs behave during the 6 months of treatment. However, due to lack of follow-up samples we were unable to measure this.

Serpin C1 was the only of the four proteins with a difference in baseline concentration between responders and non-responders to treatment with MTX in both the discovery and the replication cohort. Significantly lower protein concentrations of serpin C1 were found in the LDL EV-fraction of patients responding to MTX treatment than in non-responding patients.

Serpin C1, also known as antithrombin III (AT III), is a protein involved in inhibition of activation of protease-activated receptors (PARs) by thrombin resulting in anti-inflammatory activity [26]. MTX has been described to exert its immunomodulatory properties through inhibition or activation of a number of pathways, including the adenosine monophosphate protein kinase (AMPK) signaling pathway [44]. Part of the anti-inflammatory effect of serpin C1 exerts part of its anti-inflammatory effect through activation of the AMPK signaling pathway [45]. Upregulation of the AMPK signaling pathway leads to inhibition of nuclear factor-kB (NF-kB) signaling, consequently leading to AMPK downstream alterations in cytokine production and leukocyte activation [46]. In the group of sarcoidosis patients with an insufficient response to MTX we found higher concentrations of serpin C1 in the LDL fraction of the EVs. More serpin C1 activity could lead to upregulated signaling of the AMPK pathway. If the AMPK pathway is already upregulated in patients with higher concentrations of serpin C1 this could lead to a decrease in available ATP in the cells [47]. This ATP is needed for alteration of adenosine signaling as an effect of MTX therapy [48]. If there is less ATP available due to sustained AMPK activation due to serpin C1 activity, this could have a negative effect on the effectivity of MTX in terms of adenosine signaling.

Previous studies on EV in sarcoidosis has been performed with EVs isolated from broncho alveolar lavage fluid (BALF), resulting in EVs derived from the cells present in the airways and alveoli. From these studies, it was seen that EVs were more abundant and proteins were upregulated in BALF from patients compared to healthy controls [9, 49]. Because there is a difference in composition of immune cells in BALF versus blood there will subsequently be a difference in composition between BALF and serum EV. Nevertheless, BALF derived EV from sarcoidosis patients are capable of activating PBMCs of healthy controls in vitro as has been demonstrated by Wahlund et al. [9]. Although assessment of the pulmonary compartment may better reflect pulmonary inflammation, the use of EV derived from peripheral blood would increase clinical applicability in the future.

Our study has a number of limitations. Firstly, the study has a retrospective design; therefore, not all patient information was available, such as missing data on Scadding Stage. Secondly, the analysis was done using samples from one time point; before start of treatment. Protein concentrations may change over time, in response to disease activity. In case of serpin C1, it is not known how these levels behave prior to and after start of treatment with MTX. However, this article showed that biobanked samples of patients undergoing real world treatment are a valuable resource for EV biomarker discovery studies. Future dynamic and prospective studies are needed to further validate the value of this protein as a predictive biomarker for response to therapy.

## Conclusion

This study showed that measuring inflammatory biomarkers in EV yield results highly different from measuring the same biomarkers in the original serum sample. EVs have high potential when searching for new diagnostic or predictive biomarkers in sarcoidosis. In future studies, serpin C1 deserves special attention as we found an association between EV-concentration of serpin C1 and treatment response in patients with pulmonary sarcoidosis treated with MTX.

### Supplementary Information

** Additional file 1:** Isolation of extracellular vesicle serum fractions. LDL and HDL fractions can be obtained from serum by a DS and MnCl2 solution of DS: 0.05%, MnCl2: 0.05 M and DS: 0.65%, MnCl2: 0.2 M, respectively. For LDL fraction isolation, 25uL serum was diluted in 95 μl phosphate buffered saline (PBS) (Gibco), followed by addition of 5 μL magnetic beads (Nanomag®-D plain, 130 mm (1:25) (Micromod)). DS and MnCl2 were added into the total volume of 125 μL and were mixed. The mixture was incubated 5 min at room temperature (RT). Subsequently, the samples were placed on a bio-plex handheld magnet (Bio-Rad) and incubated 15 min at RT. The formed pellet is LDL fraction. For HDL isolation, the protocol is repeated when using 115 μL supernatant above the LDL pellet. The pellets were lysed with 125 μL Roche complete lysis-M with protease inhibitors (Roche). To remove magnetic beads and other debris, samples were centrifugated at 3200×g, 10 min. **Table S1.** Baseline characteristics of discovery cohort of sarcoidosis patients with pulmonary treatment indication and healthy controls. **Fig. S1.** Concentrations of EV biomarkers in patients with sarcoidosis treated with prednisone of the discovery cohort (*n* = 16). Figures a-d) represent protein levels measured in the LDL fraction, figures e-h) represent protein levels measured in the HDL sub fraction and figures i-l) represent protein concentrations measured in whole serum. NR = non-responder, R = responder. **Fig. S2.** Concentrations of EV biomarkers in patients with pulmonary sarcoidosis treated with MTX of the replication cohort. Figures a-d) represent protein levels measured in the LDL fraction, figures e-h) represent protein levels measured in the HDL sub fraction, and figures i-l) represent protein levels measured in whole serum. NR = non-responder, R = responder. *p < 0.05.

## Data Availability

The datasets used and/or analyzed during the current study are available from the corresponding author on reasonable request.

## Abbreviations

ACE: Angiotensin converting enzyme
AMPK: Adenosine monophosphate protein kinase
AT III: Antithrombin III
BALF: Broncho alveolar lavage fluid
DLCO: Diffusion lung of carbon monoxide
DS: Dextran sulphate
EVs: Extracellular vesicles
FVC: Forced vital capacity
HC: Healthy control
HDL: High-density lipid
LDL: Low-density lipid
MEC-U: Medical research ethics committees united
MnCl_2_: Manganese (II) chloride
MTX: Methotrexate
NF-kB: Nuclear factor-kB
sIL-2R: Soluble interleukin-2 receptor
