# Supplementary material for: Decreased serpin C1 in extracellular vesicles predicts response to methotrexate treatment in patients with pulmonary sarcoidosis
Source: Respir Res. 2024 Apr 16;25:166. doi: 10.1186/s12931-024-02809-y (PMC11020913; doi:10.1186/s12931-024-02809-y)
Supplement: Supplementary file 1 — Additional file 1: Isolation of extracellular vesicle serum fractions. LDL and HDL fractions can be obtained from serum by a DS and MnCl2 solution of DS: 0.05%, MnCl2: 0.05 M and DS: 0.65%, MnCl2: 0.2 M, respectively. For LDL fraction isolation, 25uL serum was diluted in 95 μl phosphate buffered saline (PBS) (Gibco), followed by addition of 5 μL magnetic beads (Nanomag®-D plain, 130 mm (1:25) (Micromod)). DS and MnCl2 were added into the total volume of 125 μL and were mixed. The mixture was incubated 5 min at room temperature (RT). Subsequently, the samples were placed on a bio-plex handheld magnet (Bio-Rad) and incubated 15 min at RT. The formed pellet is LDL fraction. For HDL isolation, the protocol is repeated when using 115 μL supernatant above the LDL pellet. The pellets were lysed with 125 μL Roche complete lysis-M with protease inhibitors (Roche). To remove magnetic beads and other debris, samples were centrifugated at 3200×g, 10 min. Table S1. Baseline characteristics of discovery cohort of sarcoidosis patients with pulmonary treatment indication and healthy controls. Fig. S1. Concentrations of EV biomarkers in patients with sarcoidosis treated with prednisone of the discovery cohort (n = 16). Figures a-d) represent protein levels measured in the LDL fraction, figures e-h) represent protein levels measured in the HDL sub fraction and figures i-l) represent protein concentrations measured in whole serum. NR = non-responder, R = responder. Fig. S2. Concentrations of EV biomarkers in patients with pulmonary sarcoidosis treated with MTX of the replication cohort. Figures a-d) represent protein levels measured in the LDL fraction, figures e-h) represent protein levels measured in the HDL sub fraction, and figures i-l) represent protein levels measured in whole serum. NR = non-responder, R = responder. *p < 0.05. [file 12931_2024_2809_MOESM1_ESM.zip › Serpin C1 article - TC.docx]

**Decreased Serpin C1 in extracellular vesicles predicts response to methotrexate treatment in patients with pulmonary sarcoidosis.**

Raisa Kraaijvanger^1*^, Montse Janssen Bonás^1*^, Jan C. Grutters^1,2^, Ioanna Paspali^3^, Marcel Veltkamp^1,2^, Dominique P.V. de Kleijn^3^ Coline H.M. van Moorsel^1^

1. Interstitial Lung Diseases Center of Excellence, Department of Pulmonology, St Antonius Hospital, Nieuwegein, The Netherlands
2. Division of Heart and Lungs, University Medical Center, Utrecht, The Netherlands
3. Department of Vascular Surgery, University Medical Center, Utrecht, The Netherlands

* These authors contributed equally to this work

Corresponding author: Coline van Moorsel; c.van.moorsel@antoniusziekenhuis.nl

# **Abstract**

**Background**: Sarcoidosis is a systemic granulomatous disease of unknown etiology primarily affecting the lungs. Treatment is needed when disease symptoms worsen and organ function deteriorates. In pulmonary sarcoidosis, prednisone and methotrexate (MTX) are the most common anti-inflammatory therapies. However, there is large inter-patient variability in response to treatment, and predictive response markers are currently lacking.

**Objective:** In this study, we investigated the predictive potential of biomarkers in extracellular vesicles (EVs) isolated from biobanked serum of patients with pulmonary sarcoidosis stored prior to start of therapy.

**Methods:** Protein concentrations of a four-protein test panel of inflammatory proteins were measured in a discovery (n=16) and replication (n=129) cohort of patients with sarcoidosis and 47 healthy controls. Response to therapy was defined as an improvement of the absolute score of >5% forced vital capacity (FVC) and/or >10% diffusion lung of carbon monoxide (DLCO) after 24 weeks compared to baseline (before treatment)

**Results:** Serum protein levels differed between EV fractions and serum, and between sarcoidosis cases and controls. Serpin C1 concentrations in the low density lipid particle EV fraction were lower at baseline in the group of patients with a good response to MTX treatment in both the discovery cohort (p=0.059) and in the replication cohort (p=0.032). EV Serpin C1 showed to be a significant predictor for response to treatment with MTX (OR 0.4; p=0.032).

**Conclusion:** This study shows that proteins isolated from EVs harbor a distinct signal and have potential as new predictive therapy response biomarkers in sarcoidosis.

# **Keywords**

Extracellular vesicles, sarcoidosis, biomarkers, therapy

# **Introduction**

Sarcoidosis is a systemic granulomatous disease of unknown cause mainly affecting the lungs, intrathoracic lymph nodes, eyes and skin (1). Diagnosis, monitoring, as well as predicting disease course or response to therapy is challenging in the management of patients with sarcoidosis. For decades, biomarkers such as angiotensin converting enzyme (ACE) and soluble interleukin-2 receptor (sIL-2R) have been studied to guide clinical management, unfortunately with modest sensitivity and specificity (2,3). Pharmacological treatment of sarcoidosis is initiated to prevent further specific organ damage or alleviate symptoms. Immunosuppressing and immunomodulating drugs, most often in the form of prednisone and methotrexate (MTX) are the first- and second-line choice of therapy. When initiating therapy however, it is not possible to predict the treatment response for individual patients upfront, while a significant part show no benefit from therapy. Personalized prediction of treatment response is a clinical unmet need in light of protecting patients from exposure to ineffective drugs and their side effects.

Sarcoidosis is characterized by the formation of non-caseating granulomas, persistent inflammation and activated monocytes/macrophages (4). Macrophages form the core of the granuloma, producing inflammatory cytokines and chemokines to attract lymphocytes resulting in an inflammatory environment (5). These macrophages originate from circulating bone-marrow derived monocytes which are patrolling antigen presenting cells but have a secondary function as a reservoir to replenish the macrophage pool in the tissues when needed. Several studies have highlighted an increased inflammatory status of monocytes in the blood of sarcoidosis patients (6–9). This increased inflammatory status can also be induced through extracellular vesicles (EVs) as has been described previously by Wahlund et al. (9). Furthermore, more monocytes are found in the circulation of patients with sarcoidosis and these cells have the capacity to activate other immune cells, not only through cell-cell interaction but also through monocyte derived EVs (10,11). Taken together, EVs isolated from whole blood serum may be particularly informative in sarcoidosis.

EV is an umbrella term for all vesicles found in body fluids, including exosomes, micro-vesicles and apoptotic bodies. In the last decade, it was found that EVs can functionally transfer molecules between cells (12). EVs contain nucleic acids, lipids and proteins from the releasing cells and are often referred to as liquid biopsies (13,14) reflecting the status/pathology of the releasing cell. EV proteins are often better associated with the pathology then the same freely circulating proteins in blood (15). In the search for new potential biomarkers, there has been an increased interest in EVs (16–18). Because of the relatively new stage of this emerging field of research there is a need for standardization in both methodology and technology to be able to validate the EV-associated biomarkers (19).

For this study a well-established EV-protein panel (20) was used, originally developed to predict adverse cardiovascular events. This panel consisted of four proteins, CD14, Serpin G1, Serpin C1 and Cysteine C. Although, the four proteins in this panel are mostly used in the field of cardiology, these proteins are also involved in inflammation and coagulation (21–27). Therefore, this panel of proteins could be of interest in assessment of inflammation and prediction of response to immunosuppressive treatment in sarcoidosis. The proteins were originally measured in form of a multiplex, however, for this study the proteins were measured separately to investigate the associations of the individual proteins to response to treatment.

In this exploratory study, we investigated the potential of inflammatory biomarkers derived from serum-isolated fractions of EV using a well-established panel of proteins previously validated in EVs. The goal of the present study was three-fold. First, we investigated whether differences in levels of inflammatory biomarkers exist between EV-isolates and serum. Second, we investigated whether EV-derived biomarkers differ between patients with sarcoidosis and healthy controls. Third, we assessed whether these inflammatory biomarkers predict treatment response in patients using prednisone or MTX.

# **Materials and methods**

## ***Patients***

Case and control samples were collected from the St. Antonius Hospital ILD biobank, screening all sarcoidosis patients (n=2265) for eligibility. Cases were selected based on pulmonary treatment indication (decrease in lung function, dyspnea or pulmonary fibrogenesis), treatment with prednisone or MTX, and presence of serum collected within 6 months prior to start of treatment. 98 sarcoidosis patients were treated with MTX and 69 patients treated with prednisone. All patients were adults >18 years with sarcoidosis diagnosed according to the international ATS/ERS/WASOG criteria (1).

Baseline characteristics (age at diagnosis, gender, co-morbidities as reported in the medical records), lung function, Löfgren syndrome as well as organ manifestation were recorded up to two years after start of treatment. Based on the results of the recently reported SARCORT trial (28) as well as the sarcoidosis treatment score (29), an improvement of the absolute score of >5% forced vital capacity (FVC) and/or >10% diffusion lung of carbon monoxide (DLCO) after 24 weeks compared to baseline (before treatment) classified a patient as a “responder”. All other patients were classified as “non-responder”.

This study was performed in accordance with the Declaration of Helsinki and GCP guidelines. The study was approved by the Medical research Ethics Committees United (MEC-U) of the St. Antonius Hospital (R05-08A) and written consent was obtained from all patients.

## ***Study cohorts***

First the discovery cohort was composed, a cohort of 16 prednisone and 16 MTX cases which were matched on age, sex, ethnicity and smoking history as best as possible. For each treatment, eight patients were responders and eight patients were non-responders to therapy. The replication cohort consisted of the remainder 49 prednisone and 80 MTX cases. In the replication cohort the prednisone treated group consisted of 29 responders and 20 non-responders, and the MTX treated group consisted of 42 responders and 38 non-responders. In addition, 47 healthy control samples were included.

Blood samples were collected before any treatment was given. Serum was isolated by the centrifugation of serum separator clot activator tubes at 1800g for 5 minutes and stored at −80°C in the St. Antonius Hospital BIOBANK until further use.


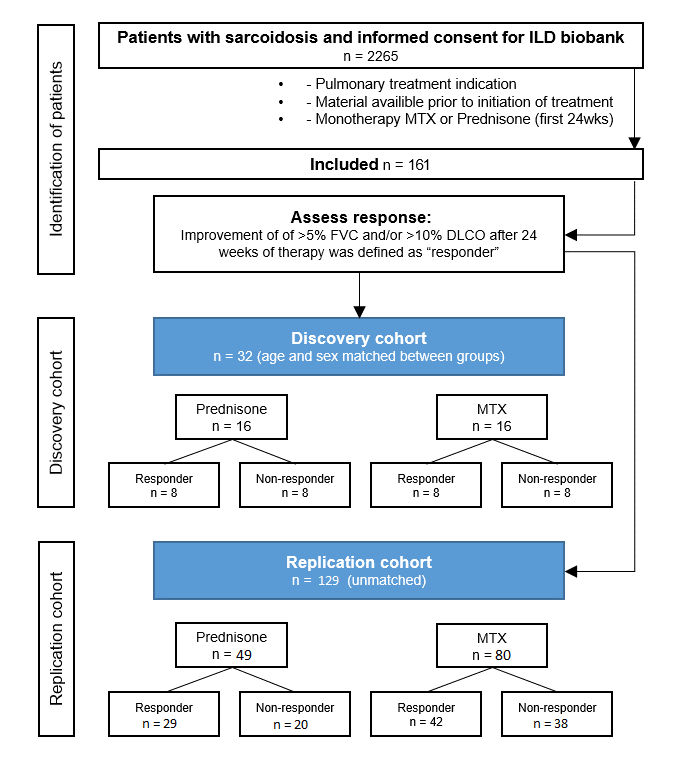


Figure 1 Flowchart showing procedure for identification of patients and distribution of patients across the discovery and replication cohort. In brief, from 2265 patients with sarcoidosis and informed consent for ILD biobank 161 patients fulfilled the criteria to be included in the study. A total of 32 sex and age matched patients were selected for the discovery cohort. The remaining 129 patients were included in the replication cohort.

## ***EV isolation***

EV fraction isolation was based on the protocol of Dekker et al. (30); however, here serum instead of plasma was used (see detailed description in the supplemental materials). EVs were isolated from 25 µL serum with the use of magnetic beads (Nanomag®-D plain, 130mm (1:25) (Micromod). For sequential isolation of the fractions Dextran Sulphate (DS) (MP Biomedicals, Illkrich, France) and Manganese (II) chloride (MnCl_2_) solution (Sigma Aldrich, St. Louis, MO, USA) were used. The presence of isolated EVs using this technique was previously confirmed with electron microscopy and western blotting (30–32). Different fractions of EVs in serum were obtained by EV co-precipitation with monolayer low-density lipid particles (LDL fraction) and with bilayer membrane vesicles high-density lipid particles (HDL fraction). Previous performed experiments have shown that relatively small EVs (± 101 nm) are present in the LDL fraction while larger particles are found in the HDL fraction (± 120 nm) (30).

## ***Protein concentration measurements***

On a 96-wells plate, LDL, HDL and whole serum were measured simultaneously. Protein concentrations in EV fractions and serum were measured in a well-established protein panel consisting of: CD14, cystatin C, serpin C1 and serpin G1 (20). Capture antibody, biotinylated detection antibody and antigen of all four proteins were purchased from R&D systems. Proteins were quantitatively analyzed by Luminex-based multiplex assay (Bio-Rad, Austin, USA). All EV protein levels were corrected for total amount of protein.

## ***Statistical analysis***

Non-parametric Mann-Whitney U test was used for non-normally distributed data. Categorical variables were compared using Chi-Squared and Fisher’s exact test, where appropriate. Log transformed values of the protein levels were used to reduce the effect of skewness in the distribution of the protein-levels. To calculate the odds ratio and enable the direct comparison between different proteins, EV-protein levels were converted into standardized units, or the z-score, by using the observed value minus the mean value, divided by the standard deviation. To investigate the relationship between the protein levels and response to therapy, a logistic regression model was used with the outcome “response to medication”. Spearman’s rho correlations were calculated to assess direct relationships between protein concentrations and other parameters. For the analysis in the discovery cohort p-values <0.1 were considered of significant interest, for the analysis with the replication cohort and the combined cohort p-values <0.05 were considered significant.

# **Results**

### ***Discovery cohort***

### ***Baseline characteristics***

Baseline characteristics of the age, sex, ethnicity and smoking history matched discovery cohort of the patient groups treated with MTX or prednisone are shown in table 1. For the MTX group the DLCO %pred was significantly lower in the responding group (p=0.010).

Table 1 **Baseline characteristics of discovery cohort of sarcoidosis patients with pulmonary treatment indication**.

| **Parameter** | | **Prednisone treated group** | | **Methotrexate treated group** | |
| --- | --- | --- | --- | --- | --- |
|  |  | **Non-responding**  **(n=8)** | **Responding**  **(n=8)** | **Non-responding (n=8)** | **Responding**  **(n=8)** |
| **Age^a^ (years)** | | 43.3 ± 6.7 | 37.4 ± 8.5 | 48.3 ± 7.0 | 52.6 ± 13.0 |
| **Male sex** | | 5 (62.5) | 5 (62.5) | 5 (62.5) | 5 (62.5) |
| **Ever smoker** | | 2 (25.0) | 2 (25.0) | 2 (25.0) | 2 (25.0) |
| **Caucasian** | | 8 (100) | 8 (100) | 8 (100) | 8 (100) |
| **Lofgren syndrome** | | 0 (0.0) | 1 (12.5) | 1 (12.5) | 0 (0.0) |
| **Scadding stage^b^**  **0/I/II/III/IV** | | 1/2/2/1/2  (12.5/25/25/12.5/25) | 1/1/3/1/2  (12.5/12.5/37.5/12.5/25) | 0/1/5/0/2  (0/12.5/62.5/0/25) | 0/1/4/1/2  (0/12.5/50/12.5/25) |
| **Lung function^b^** | |  |  |  |  |
|  | FVC (%) | 94.5 ± 28.5 | 81.8 ± 24.2 | 102.7 ± 11.6 | 89.0 ±21.8 |
|  | DLCO (%) | 75.8 ± 25.0 | 66.1 ± 13.2 | 86.1 ± 12.6* | 55.5 ± 13.1* |
| **Extra-pulmonary involvement** | | |  |  |  |
|  | Lymph nodes | 5 (62.5) | 4 (50.0) | 5 (62.5) | 4 (50.0) |
|  | Skin | 0 (0.0) | 1 (12.5) | 0 (0.0) | 1 (12.5) |
|  | Liver | 0 (0.0) | 0 (0.0) | 1 (12.5) | 0 (0.0) |
|  | Spleen | 1 (12.5) | 1 (12.5) | 1 (12.5) | 0 (0.0) |
|  | SFN | 0 (0.0) | 0 (0.0) | 0 (0.0) | 0 (0.0) |

Data is shown as whole numbers and percentages between brackets. Response to treatment was based on improvement in lung function (FVC %pred >10% or DLCO %pred >10%) after 6 months of treatment. Age, lung function, and biomarkers are shown as mean ± SD. ^a^Age at time of blood withdrawal. ^b^lung function and scadding stage were measured before start of treatment. Scadding stages: 0 = Normal chest radiograph; I = Bilateral hilar lymphadenopathy (BHL); II = BHL with pulmonary infiltrates; III = pulmonary infiltrates without BHL; IV = fibrosis. SFN: Small fiber neuropathy. DLCO (%) was significantly lower in patients responding to MTX therapy. *p<0.05

## ***Differences in protein levels between EV and serum in sarcoidosis***

The proteins from the protein test panel were measured in the EV-fractions LDL, HDL and in whole serum. Figure 2 shows the concentrations of the different proteins measured in the discovery cohort (n=32). Compared with EV-fractions, whole serum protein concentrations were higher for CD14, Cystatin C and Serpin C1, but not for Serpin G1. Furthermore, protein concentrations for Serpin G1 and Serpin C1 were lower in the HDL fraction than in the LDL faction.


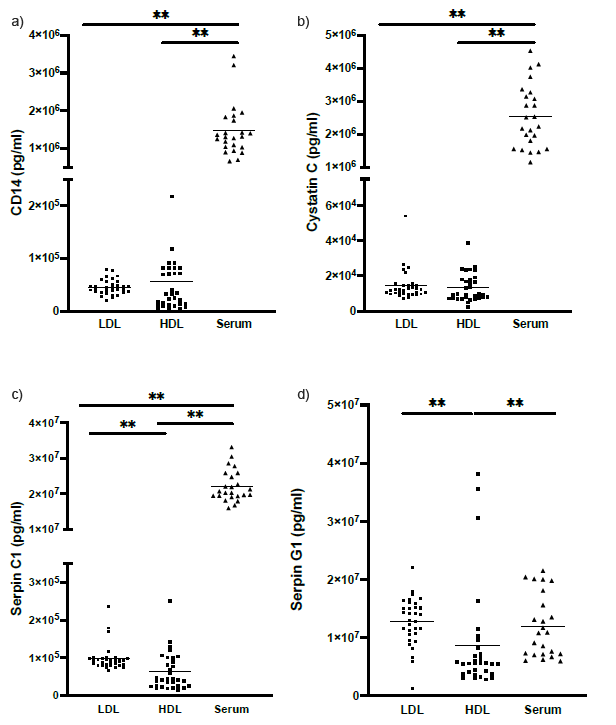


Figure 2. Concentrations of proteins measured in different EV fractions (LDL and HDL) and in whole serum in the sarcoidosis discovery cohort (n=32). For all proteins except serpin G1 serum concentrations were higher compared to EV-fractions (LDL and HDL). For both serpin G1 and C1 there was a difference between concentrations in LDL and HDL. LDL = low-density lipid, HDL = high-density lipid. **p<0.005

## ***EV-derived proteins are higher in sarcoidosis than in controls***

The proteins from the protein test panel measured in the discovery cohort were compared to protein levels in healthy controls (HC) (n=47). Baseline characteristics are shown in supplementary table S1. The concentration of proteins was significantly higher in sarcoidosis patients compared to HC when measured in whole serum (figure 3). For the HDL EV-fraction only serpin C1 was significantly different between patients and HC. For the LDL fraction, a significant difference was observed for all proteins of the test panel. When compared to healthy controls, the concentrations of CD14 and cystatin C were higher in the LDL fractions of patients while concentrations of both serpin proteins were lower in LDL fractions.


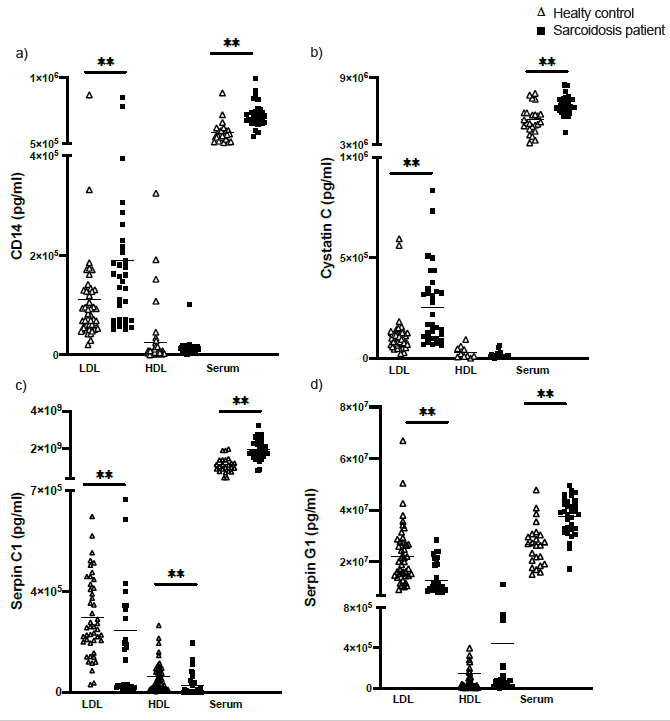


Figure 3. Concentrations of proteins from EV-protein test panel. Light triangles represent healthy controls and dark squares represent sarcoidosis patients from the discovery cohort. a) Concentrations of CD14; b) concentrations of cystatin C; c) concentrations of serpin C1; and d) concentrations of serpin G1 measured in EV fractions LDL and HDL and in whole serum. LDL = low-density lipid, HDL = high-density lipid. **p<0.005

***Response to treatment***

In the prednisone discovery cohort no differences were found in protein concentrations between responders and non-responders in either LDL, HDL fractions or serum.

In the MTX discovery cohort, concentrations of serpin C1 were lower (p=0.059) in the group of responders while concentrations of CD14 were significantly higher in the group of patients classified as responders (p=0.014) in the LDL fraction. In the HDL fraction, cystatin C concentrations were lower in the group of patients classified as non-responders (p=0.027) (figure 4). No difference was found in serum protein levels of responders and non-responders (supplementary figure S1).


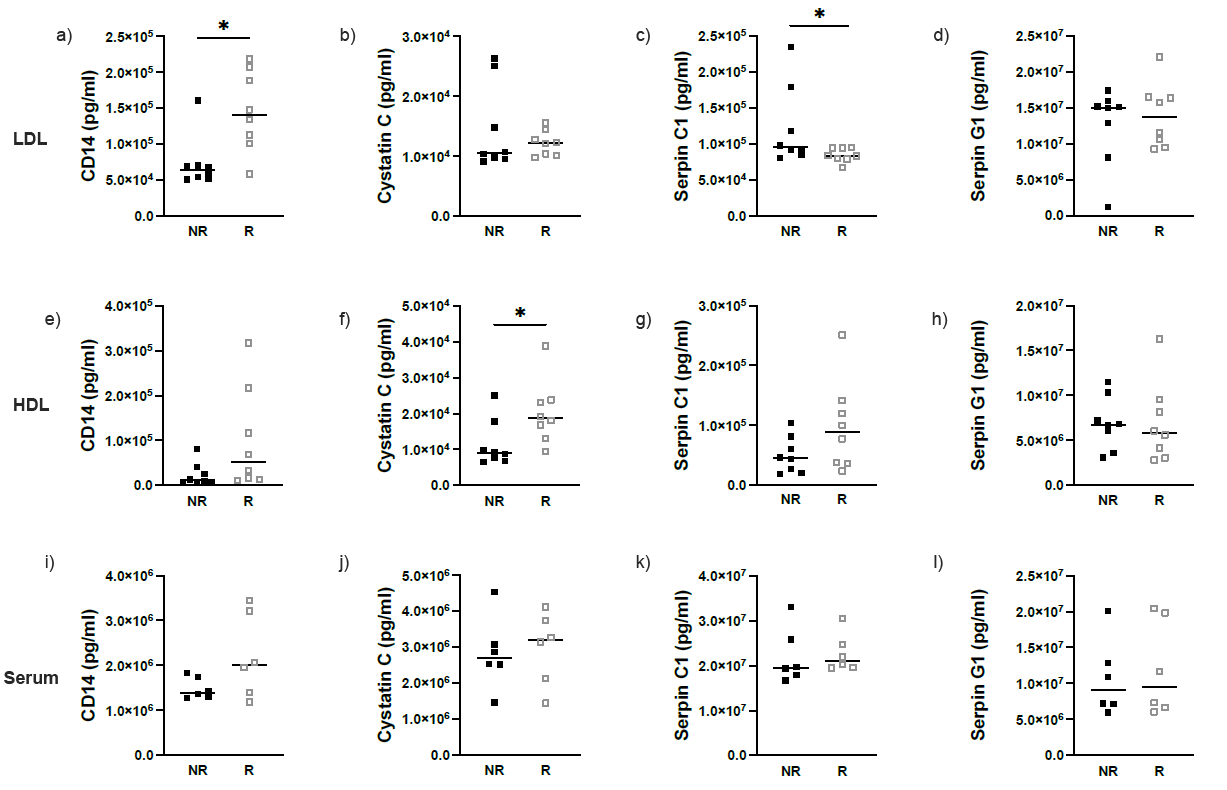


Figure 4. Concentrations of proteins from EV-protein test panel in patients with sarcoidosis treated with methotrexate of the discovery cohort (n=16). Figures a-d) represents EV-proteins measured in the LDL fraction, figures e-h) EV-proteins measured in the HDL fraction and figures i-l) proteins measured in whole serum. Filled squares represent non-responders (NR) and open squares represent responders (R) to treatment with methotrexate. *p<0.1

### ***Replication cohort***

### ***Baseline characteristics***

In the second phase of the study, the EV proteins were quantified in a larger replication cohort of 80 sarcoidosis patients treated with methotrexate and 49 sarcoidosis patients treated with prednisone. Baseline characteristics are shown in table 2. As with the discovery cohort, all included patients had a pulmonary treatment indication.

Table 2. **Baseline characteristics of the replication cohort of sarcoidosis patients with pulmonary treatment indication**.

| **Parameter** | | **Prednisone treated group** | | **Methotrexate treated group** | |
| --- | --- | --- | --- | --- | --- |
|  |  | **Non-responding**  **(n=20)** | **Responding**  **(n=29)** | **Non-responding (n=38)** | **Responding**  **(n=42)** |
| **Age^a^ (years)** | | 43.6 ± 10.0 | 43.5 ± 9.6 | 49.0 ± 10.0 | 47.9 ± 10.9 |
| **Male sex** | | 14 (70.0) | 21 (72.4) | 23 (71.9) | 25 (59.5) |
| **Ever smoker** | | 5 (31.3) | 10 (47.6) | 15 (60.0) | 18 (52.9) |
| **Caucasian** | | 18 (90.0) | 26 (92.9) | 28 (90.3) | 36 (90.0) |
| **Lofgren syndrome** | | 0 (0.0) | 2 (7.1) | 2 (6.3) | 2 (4.8) |
| **Scadding stage^b^**  **0/I/II/III/IV** | | 0/2/12/1/4  (0/11/63/5/21) | 0/5/14/1/7  (0/19/52/4/26) | 2/3/11/1/12  (7/10/38/3/4130) | 2/7/18/0/7  (6/20/53/0/21) |
| **Lung function^b^** | |  |  |  |  |
|  | FVC (%) | 94.5 ± 19.1 | 83.5 ± 20.4 | 97.1 ± 22.2 | 91.1 ± 23.1 |
|  | DLCO (%) | 70.3 ± 16.3 | 66.6 ± 17.8 | 72.3 ± 15.0 | 67.7 ± 15.5 |
| **Extra-pulmonary involvement** | | |  |  |  |
|  | Lymph nodes | 12 (60.0) | 16 (55.2) | 22 (68.8) | 28 (66.7) |
|  | Skin | 0 (0.0) | 3 (10.3) | 6 (18.8) | 4 (9.5) |
|  | Liver | 1 (5.0) | 1 (3.4) | 5 (15.6) | 6 (14.3) |
|  | Spleen | 5 (25.0) | 5 (17.2) | 7 (21.9) | 7 (16.7) |
|  | SFN | 4 (20.0) | 2 (6.9) | 5 (15.6) | 3 (7.1) |

Data is shown as whole numbers and percentages between brackets. Response to treatment was based on improvement in lung function (FVC %pred >10% or DLCO %pred >10%) after 6 months of treatment. Age, lung function, and biomarkers are shown as mean ± SD. ^a^Age at time of blood withdrawal. ^b^lung function and scadding stage were measured before start of treatment. Scadding stages: 0 = Normal chest radiograph; I = Bilateral hilar lymphadenopathy (BHL); II = BHL with pulmonary infiltrates; III = pulmonary infiltrates without BHL; IV = fibrosis. SFN: Small fiber neuropathy.

Supplementary figure S2 shows the concentrations of all proteins measured in the replication cohort. Regarding the proteins of interest identified in the discovery cohort, serpin C1 concentrations in the LDL fraction were significantly lower in the methotrexate responder than in the non-responder group of the replication cohort (p=0.032; figure 5a). Regarding CD14 in LDL and cystatin C in HDL, however, no difference between responders and non-responders was found (p=0.091 and p=0.215, respectively, Figure 5b,c).


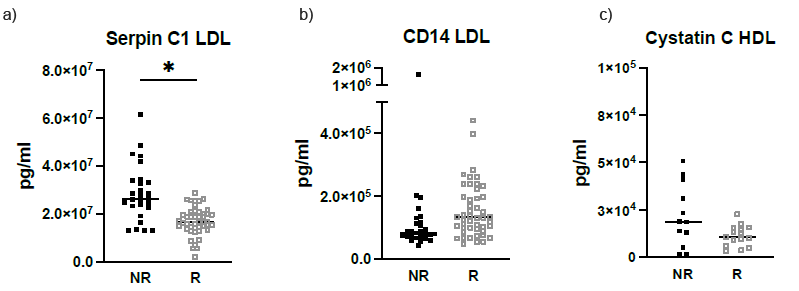


Figure 5. Concentrations of EV proteins in patients with sarcoidosis treated with methotrexate of the replication cohort (n=82), measured in different EV fractions as well as in whole serum. Figure a) represents concentrations of Serpin C1 in the LDL fraction; figure b) concentrations of CD14 in the LDL fraction, and figure c) concentrations of cystatin C in the HDL fraction in non-responders and responders to treatment with methotrexate. Serpin C1 concentrations were significantly higher in non-responders than in responders (p=0.032). Black filled squares represent non-responders (NR) and gray open squares represent responders (R) to treatment with methotrexate. *p<0.05

## ***EV proteins to predict response to MTX***

To determine the predictive value of serpin C1 for treatment response prediction we combined the MTX discovery and replication cohorts for further analysis. Values were log-transformed to stabilize the variance. Logistic regression revealed that serpin C1, when measured in the LDL sub fraction, was a significant predictor for response to treatment (OR 0.42 [95%CI 0.19 – 0.93] p = 0.032).

To investigate if there was a direct relation, Spearman’s rank correlations were computed to assess the relation between change in FVC 6 months after initiation of MTX therapy and protein concentrations at baseline. There was a positive correlation between change in FVC and CD14 concentrations in the LDL fraction, (R=0.35; p=0.004). None of the other proteins directly correlated with change in FVC and no correlation was found for change in DLCO.

# **Discussion**

In this study, we showed that concentrations of proteins in serum and in EVs fractions isolated from serum can differ significantly from each other and that this depends on the analyzed protein. Second, we found that levels of proteins in EV fractions differed significantly between sarcoidosis and healthy controls. Third, we showed that EV proteins, and particularly serpin C1 in the LDL EV-fraction is significantly lower at baseline in the group of patients that responded to treatment with MTX, which we verified in a second cohort. Baseline serpin C1 was shown to be a marker with predictive value of response to MXT therapy.

Our data demonstrates that measuring proteins specifically in EV can provide novel information in sarcoidosis research compared to measuring the same proteins in serum alone. Although EVs have the disadvantage of an extra isolation step, they do also have advantages such as stability, easy accessibility and minimal sample volumes (18). Furthermore, EVs are increasingly recognized to contain proteins involved in cellular processes directly linked to disease pathogenesis (33).

The difference in protein expression between patients and healthy controls is more evident in the LDL fraction than in the HDL fraction. A possible explanation for this observation lies in the difference in composition of the vesicles, HDL vesicles are larger and are suggested to contain more proteins from the originating cells (34,35). While the LDL fraction consist of smaller EVs including exosomes which are released by activated immune cells and have the capacity to activate other immune cells. The overly active immune system in sarcoidosis patients could lead to an increased release in exosomes eventually resulting in higher EV protein concentrations in patients versus healthy controls (36). Protein concentrations of pro-inflammatory cytokines CD14 and Cystatin C have been shown to be elevated in multiple inflammatory disorders (37,38) and were also elevated in the LDL fraction in sarcoidosis patients compared to healthy controls. Both serpin C1 and G1 were elevated in healthy controls. These serpins exert anti-inflammatory properties (21,39). However, as previous research describes a decrease in EV numbers with advancing age (40). We also have to take into account the possibility of an age effect on the differences in EV-protein concentrations between patients and healthy controls, since the healthy controls were significantly younger. When comparing the concentrations of the proteins with the age of the healthy control subject, none of the proteins correlated with age resulting in a minimal impact of age on the difference in EV-protein concentrations between patients and healthy controls.

In light of diagnostic potential of these biomarkers, there was no added value of EV over serum since all four proteins also differed significantly between patients and healthy controls in serum. Further analysis, using serum samples from time of diagnosis from patients with sarcoidosis and differential diagnoses are needed to determine the diagnostic potential of the markers. The EV proteins in our panel are general inflammation markers, found on the cell surface, shed from the cells or actively involved in cellular inflammatory processes. Therefore, it is not surprising that we found a difference in protein concentrations between healthy controls and patients. However, an interesting finding was made for serpin C1. In the LDL and HDL EV-fractions, serpin C1 concentrations were lower in patients than in controls, while in whole serum, the serpin C1 concentration was higher in patients than in controls. The effect of serpin C1 is different in the circulation than as a cellular surface maker, where it has an increased anti-inflammatory effect. As previously suggested, more serpin C1 may have been shed in patients with sarcoidosis compared to healthy controls (41), which corresponds to our increased levels in the serum of patients.

Further aim of investigation was to assess whether protein concentrations in EV could have a predictive value for response to therapy. To assess this, patients receiving either prednisone or MTX were divided in responders and non-responders, based on change in lung function. The protein test panel revealed no difference in EV-fractions between responders and non-responders in the group of patients treated with prednisone. For the patient group treated with MTX, Serpin C1 concentrations were significantly lower in the group of patients responding to treatment while CD14 concentrations were higher in the responder group, unfortunately for CD14 the difference between responders and non-responder was no longer significant. In addition, we found a positive correlation between CD14 concentrations at baseline and change in FVC over the course of 6 months of MTX treatment (p=0.004). This protein was previously identified as an EV marker for sarcoidosis by Futami et al., who described CD14 levels in EVs to be significantly increased in patients with sarcoidosis and showed that CD14 was up-regulated in the process of granuloma formation (42). In MTX treated patients with RA a positive correlation has been described between the concentration of soluble CD14 (sCD14) and response to MTX, RA patients with the highest sCD14 concentration at baseline responded best to MTX treatment. Our findings suggest a similar effect as responders have the highest concentration of CD14 in the vesicles at baseline and this correlates with highest change in FVC (43). In future, it would be interesting to see how the CD14 concentration in EVs behave during the 6 months of treatment. However, due to lack of follow-up samples we were unable to measure this.

Serpin C1 was the only of the four proteins with a difference in baseline concentration between responders and non-responders to treatment with MTX in both the discovery and the replication cohort. Significantly lower protein concentrations of serpin C1 were found in the LDL EV-fraction of patients responding to MTX treatment than in non-responding patients.

Serpin C1, also known as antithrombin III (AT III), is a protein involved in inhibition of activation of protease-activated receptors (PARs) by thrombin resulting in anti-inflammatory activity (26). MTX has been described to exert its immunomodulatory properties through inhibition or activation of a number of pathways, including the adenosine monophosphate protein kinase (AMPK) signaling pathway (44). Part of the anti-inflammatory effect of serpin C1 exerts part of its anti-inflammatory effect through activation of the AMPK signaling pathway (45). Upregulation of the AMPK signaling pathway leads to inhibition of nuclear factor-kB (NF-kB) signaling, consequently leading to AMPK downstream alterations in cytokine production and leukocyte activation (46). In the group of sarcoidosis patients with an insufficient response to MTX we found higher concentrations of serpin C1 in the LDL fraction of the EVs. More serpin C1 activity could lead to upregulated signaling of the AMPK pathway. If the AMPK pathway is already upregulated in patients with higher concentrations of serpin C1 this could lead to a decrease in available ATP in the cells (47). This ATP is needed for alteration of adenosine signaling as an effect of MTX therapy (48). If there is less ATP available due to sustained AMPK activation due to serpin C1 activity, this could have a negative effect on the effectivity of MTX in terms of adenosine signaling.

Previous studies on EV in sarcoidosis has been performed with EVs isolated from broncho alveolar lavage fluid (BALF), resulting in EVs derived from the cells present in the airways and alveoli. From these studies, it was seen that EVs were more abundant and proteins were upregulated in BALF from patients compared to healthy controls (9,49). Because there is a difference in composition of immune cells in BALF versus blood there will subsequently be a difference in composition between BALF and serum EV. Nevertheless, BALF derived EV from sarcoidosis patients are capable of activating PBMCs of healthy controls in vitro as has been demonstrated by Wahlund et al (9). Although assessment of the pulmonary compartment may better reflect pulmonary inflammation, the use of EV derived from peripheral blood would increase clinical applicability in the future.

Our study has a number of limitations. Firstly, the study has a retrospective design; therefore, not all patient information was available, such as missing data on Scadding Stage. Secondly, the analysis was done using samples from one time point; before start of treatment. Protein concentrations may change over time, in response to disease activity. In case of serpin C1, it is not known how these levels behave prior to and after start of treatment with MTX. However, this article showed that biobanked samples of patients undergoing real world treatment are a valuable resource for EV biomarker discovery studies. Future dynamic and prospective studies are needed to further validate the value of this protein as a predictive biomarker for response to therapy.

# **Conclusion**

This study showed that measuring inflammatory biomarkers in EV yield results highly different from measuring the same biomarkers in the original serum sample. EVs have high potential when searching for new diagnostic or predictive biomarkers in sarcoidosis. In future studies, serpin C1 deserves special attention as we found an association between EV-concentration of serpin C1 and treatment response in patients with pulmonary sarcoidosis treated with MTX.

# **List of abbreviations**

ACE Angiotensin converting enzyme

AMPK Adenosine monophosphate protein kinase

AT III Antithrombin III

BALF Broncho alveolar lavage fluid

DLCO Diffusion lung of carbon monoxide

DS Dextran Sulphate

EVs Extracellular vesicles

FVC Forced vital capacity

HC Healthy control

HDL High-density lipid

LDL Low-density lipid

MEC-U Medical research Ethics Committees United

MnCl_2_ Manganese (II) chloride

MTX Methotrexate

NF-kB Nuclear factor-kB

sIL-2R Soluble interleukin-2 receptor

# **Funding**

This study is funded by the Lung Foundation (nr 5.1.19.035), the Netherlands and by the St. Antonius Onderzoeksfonds (nr 21.07). Funders had no involvement in the study design; in the collection, analysis and interpretation of the data; in the writing of the report or in the decision to submit the paper for publication.

# **Ethics approval and consent to participate**

This study was performed in accordance with the Declaration of Helsinki and GCP guidelines. The study was approved by the Medical research Ethics Committees United (MEC-U) (R14.023) the local institutional review board (Z.19.004) of the St Antonius Hospital.

# **Consent for publication**

Not applicable

# **Conflicts of interest**

The authors declare that they have no competing interests.

# **Availability of data and materials**

The datasets used and/or analyzed during the current study are available from the corresponding author on reasonable request.

# **Authors' contributions**

RK and MJB contributed to the design of the study, data acquisition and analysis, statistical analysis, drafting, and editing of the manuscript. IP contributed to data acquisition. JG supervised the project and edited the manuscript. MV participated in the design of the study, data interpretation, supervision and editing the manuscript. DdK supervised part of the project and edited the manuscript. CvM designed the study, supervised the project and edited the manuscript.

# **Acknowledgement**

Not applicable

# **References**

1. Costabel U, Hunninghake GW. ATS/ERS/WASOG statement on sarcoidosis. Sarcoidosis Statement Committee. American Thoracic Society. European Respiratory Society. World Association for Sarcoidosis and Other Granulomatous Disorders. *Eur Respir J* (1999) 14:735–7. doi: 10.1034/j.1399-3003.1999.14d02.x

2. Korenromp IHE, Maier LA, Grutters JC. Sarcoidosis: serum and imaging biomarkers. *Eur Respir monogr* (2022) 96:107–121. doi: 10.1183/2312508X.10031720

3. Kraaijvanger R, Janssen Bonás M, Vorselaars A, Veltkamp M. Biomarkers in the Diagnosis and Prognosis of Sarcoidosis: Current Use and Future Prospects. *Front Immunol* (2020) 11:1–17. doi: 10.3389/fimmu.2020.01443

4. Chopra A, Kalkanis A, Judson MA. Biomarkers in sarcoidosis. *Expert Rev Clin Immunol* (2016) 12:1191–1208. doi: 10.1080/1744666X.2016.1196135

5. Cinetto F, Scarpa R, Dell’Edera A, Jones MG. Immunology of sarcoidosis: old companions, new relationships. *Curr Opin Pulm Med* (2020) 26:535–543. doi: 10.1097/MCP.0000000000000711

6. Fraser SD, Hart SP. Monocytes and macrophages in chronic sarcoidosis pathology. *Eur Respir J* (2019) 54:1901626. doi: 10.1183/13993003.01626-2019

7. Fraser SD, Sadofsky LR, Kaye PM, Hart SP. Reduced expression of monocyte CD200R is associated with enhanced proinflammatory cytokine production in sarcoidosis. *Sci Rep* (2016) doi: 10.1038/srep38689

8. Dubaniewicz A, Typiak M, Wybieralska M, Szadurska M, Nowakowski S, Staniewicz-Panasik A, Rogoza K, Sternau A, Deeg P, Trzonkowski P. Changed phagocytic activity and pattern of Fcγ and complement receptors on blood monocytes in sarcoidosis. *Hum Immunol* (2012) 73:788–794. doi: 10.1016/j.humimm.2012.05.005

9. Wahlund CJE, Gucluler Akpinar G, Steiner L, Ibrahim A, Bandeira E, Lepzien R, Lukic A, Smed-Sörensen A, Kullberg S, Eklund A, et al. Sarcoidosis exosomes stimulate monocytes to produce pro-inflammatory cytokines and CCL2. *Sci Rep* (2020) 10: doi: 10.1038/s41598-020-72067-7

10. Roig-Arcos J, López-Malo D, Díaz-Llopis M, Romero FJ. Exosomes derived from stimulated monocytes promote endothelial dysfunction and inflammation in vitro. *Ann Transl Med* (2017) 5:258–258. doi: 10.21037/atm.2017.03.101

11. Lepzien R, Nie M, Czarnewski P, Liu S, Yu M, Ravindran A, Kullberg S, Eklund A, Grunewald J, Smed-Sörensen A. Pulmonary and blood dendritic cells from sarcoidosis patients more potently induce IFNγ-producing Th1 cells compared with monocytes. *J Leukoc Biol* (2022) 111:857–866. doi: 10.1002/JLB.5A0321-162R

12. Dickhout A, Koenen RR. Extracellular Vesicles as Biomarkers in Cardiovascular Disease; Chances and Risks. *Front Cardiovasc Med* (2018) 5:113. doi: 10.3389/fcvm.2018.00113

13. Fujita Y, Kosaka N, Araya J, Kuwano K, Ochiya T. Extracellular vesicles in lung microenvironment and pathogenesis. *Trends Mol Med* (2015) 21:533–542. doi: 10.1016/j.molmed.2015.07.004

14. Maas SLN, Breakefield XO, Weaver AM. Extracellular Vesicles: Unique Intercellular Delivery Vehicles. *Trends Cell Biol* (2017) 27:172–188. doi: 10.1016/j.tcb.2016.11.003

15. Buzas EI. The roles of extracellular vesicles in the immune system. *Nat Rev Immunol* (2023) 23:236–250. doi: 10.1038/s41577-022-00763-8

16. Carnino JM, Lee H. Extracellular vesicles in respiratory disease. *Adv Clin Chem* (2022) 108:105–127. doi: 10.1016/BS.ACC.2021.07.008

17. Raposo G, Stoorvogel W. Extracellular vesicles: Exosomes, microvesicles, and friends. J Cell Biol (2013). doi: 10.1083/jcb.201211138

18. Théry C, Witwer KW, Aikawa E, Alcaraz MJ, Anderson JD, Andriantsitohaina R, Antoniou A, Arab T, Archer F, Atkin-Smith GK, et al. Minimal information for studies of extracellular vesicles 2018 (MISEV2018): a position statement of the International Society for Extracellular Vesicles and update of the MISEV2014 guidelines. *J Extracell Vesicles* (2018) 7:1535750. doi: 10.1080/20013078.2018.1535750

19. Coumans FAW, Brisson AR, Buzas EI, Dignat-George F, Drees EEE, El-Andaloussi S, Emanueli C, Gasecka A, Hendrix A, Hill AF, et al. Methodological guidelines to study extracellular vesicles. *Circ Res* (2017) 120:1632–1648. doi: 10.1161/CIRCRESAHA.117.309417

20. Verwer MC, Mekke JM, Timmerman N, Van Der Pol QY, Frissen C, Pasterkamp G, De Borst GJ, Hazenberg CEVB, De Kleijn DPV. Plasma extracellular vesicle SerpinG1 and CD14 levels are associated with MACE and MALE in patients undergoing femoral endarterectomy. *Eur J Vasc Endovasc Surg* (2022)1–9. doi: 10.1016/j.ejvs.2022.10.045

21. Oelschläger C, Römisch J, Staubitz A, Stauss H, Leithäuser B, Tillmanns H, Hölschermann H. Antithrombin III inhibits nuclear factor κB activation in human monocytes and vascular endothelial cells. *Blood* (2002) 99:4015–4020. doi: 10.1182/blood.V99.11.4015

22. Pforte A, Schiessler A, Gais P, Beer B, Ehlers M, Schutt C, Ziegler- Heitbrock HWL. Expression of CD14 correlates with lung function impairment in pulmonary sarcoidosis. *Chest* (1994) 105:349–354. doi: 10.1378/chest.105.2.349

23. Orlowski GM, Colbert JD, Sharma S, Bogyo M, Robertson SA, Rock KL. Multiple Cathepsins Promote Pro–IL-1β Synthesis and NLRP3-Mediated IL-1β Activation. *J Immunol* (2015) 195:1685–1697. doi: 10.4049/jimmunol.1500509

24. Bergantini L, d’Alessandro M, Cameli P, Carleo A, Landi C, Vietri L, Lanzarone N, Pieroni M, Sestini P, Bargagli E. Antithrombin III as predictive indicator of survival in idiopathic pulmonary fibrosis (IPF) patients treated with nintedanib: a preliminary study. *Intern Med J* (2021) 51:705–711. doi: 10.1111/imj.14768

25. Meyer-Siegler KL, Cox J, Leng L, Bucala R, Vera PL. Macrophage migration inhibitory factor anti-thrombin III complexes are decreased in bladder cancer patient serum: Complex formation as a mechanism of inactivation. *Cancer Lett* (2010) 290:49–57. doi: 10.1016/j.canlet.2009.08.025

26. Davis AE, Mejia P, Lu F. Biological activities of C1 inhibitor. *Mol Immunol* (2008) 45:4057–63. doi: 10.1016/j.molimm.2008.06.028

27. Lubbers R, Sutherland JS, Goletti D, de Paus RA, Dijkstra DJ, van Moorsel CHM, Veltkamp M, Vestjens SMT, Bos WJW, Petrone L, et al. Expression and production of the SERPING1-encoded endogenous complement regulator C1-inhibitor in multiple cohorts of tuberculosis patients. *Mol Immunol* (2020) 120:187–195. doi: 10.1016/j.molimm.2020.02.006

28. Dhooria S, Sehgal IS, Agarwal R, Muthu V, Prasad KT, Dogra P, Debi U, Garg M, Bal A, Gupta N, et al. High-dose (40 mg) versus low-dose (20 mg) prednisolone for treating sarcoidosis: a randomised trial (SARCORT trial). *Eur Respir J* (2023) 62: doi: 10.1183/13993003.00198-2023

29. Baughman RP, Tillinger M, Qin Y, Sweiss N, Lower EE. A composite score to assess treatment response in pulmonary sarcoidosis: The sarcoidosis treatment Score (STS). *Sarcoidosis Vasc Diffus Lung Dis* (2019) 36:86–88. doi: 10.36141/svdld.v36i1.7748

30. Dekker M, Waissi F, van Bennekom J, Silvis MJM, Timmerman N, Bank IEM, Walter JE, Mueller C, Schoneveld AH, Schiffelers RM, et al. Plasma extracellular vesicle proteins are associated with stress-induced myocardial ischemia in women presenting with chest pain. *Sci Rep* (2020) 10:1–8. doi: 10.1038/s41598-020-69297-0

31. Wang JW, Zhang YN, Sze SK, van de Weg SM, Vernooij F, Schoneveld AH, Tan SH, Versteeg HH, Timmers L, Lam CSP, et al. Lowering low-density lipoprotein particles in plasma using dextran sulphate co-precipitates procoagulant extracellular vesicles. *Int J Mol Sci* (2018) doi: 10.3390/ijms19010094

32. Zhang YN, Vernooij F, Ibrahim I, Ooi S, Gijsberts CM, Schoneveld AH, Sen KW, Den Ruijter HM, Timmers L, Richards AM, et al. Extracellular vesicle proteins associated with systemic vascular events correlate with heart failure: An observational study in a dyspnoea cohort. *PLoS One* (2016) doi: 10.1371/journal.pone.0148073

33. Han C, Yang J, Sun J, Qin G. Extracellular vesicles in cardiovascular disease: Biological functions and therapeutic implications. *Pharmacol Ther* (2022) 233:1–44. doi: 10.1016/j.pharmthera.2021.108025

34. Abels ER, Breakefield XO. Introduction to Extracellular Vesicles: Biogenesis, RNA Cargo Selection, Content, Release, and Uptake. *Cell Mol Neurobiol* (2016) 36:301–312. doi: 10.1007/s10571-016-0366-z

35. Carnino JM, Lee H, Jin Y. Isolation and characterization of extracellular vesicles from Broncho-alveolar lavage fluid: a review and comparison of different methods. *Respir Res* (2019) 20:240. doi: 10.1186/s12931-019-1210-z

36. Hazrati A, Soudi S, Malekpour K, Mahmoudi M, Rahimi A, Hashemi SM, Varma RS. Immune cells-derived exosomes function as a double-edged sword: role in disease progression and their therapeutic applications. *Biomark Res* (2022) 10:1–25. doi: 10.1186/s40364-022-00374-4

37. Zi M, Xu Y. Involvement of cystatin C in immunity and apoptosis. *Immunol Lett* (2018) 196:80–90. doi: 10.1016/j.imlet.2018.01.006

38. Wu Z, Zhang Z, Lei Z, Lei P. CD14: Biology and role in the pathogenesis of disease. *Cytokine Growth Factor Rev* (2019) 48:24–31. doi: 10.1016/j.cytogfr.2019.06.003

39. Cicardi M, Zingale L, Zanichelli A, Pappalardo E, Cicardi B. C1 inhibitor: Molecular and clinical aspects. *Springer Semin Immunopathol* (2005) 27:286–298. doi: 10.1007/s00281-005-0001-4

40. Eitan E, Green J, Bodogai M, Mode NA, Bæk R, Jørgensen MM, Freeman DW, Witwer KW, Zonderman AB, Biragyn A, et al. Age-Related Changes in Plasma Extracellular Vesicle Characteristics and Internalization by Leukocytes. *Sci Rep* (2017) 7:1–14. doi: 10.1038/s41598-017-01386-z

41. Radeghieri A, Alacqua S, Zendrini A, Previcini V, Todaro F, Martini G, Ricotta D, Bergese P. Active antithrombin glycoforms are selectively physiosorbed on plasma extracellular vesicles. *J Extracell Biol* (2022) 1: doi: 10.1002/jex2.57

42. Futami Y, Takeda Y, Koba T, Narumi R, Nojima Y, Ito M, Nakayama M, Ishida M, Yoshimura H, Naito Y, et al. Identification of CD14 and lipopolysaccharide-binding protein as novel biomarkers for sarcoidosis using proteomics of serum extracellular vesicles. *Int Immunol* (2022) 34:327–340. doi: 10.1093/intimm/dxac009

43. Fuentelsaz-Romero S, Barrio-Alonso C, García Campos R, Torres Torresano M, Muller IB, Triguero-Martínez A, Nuño L, Villalba A, García-Vicuña R, Jansen G, et al. The Macrophage Reprogramming Ability of Antifolates Reveals Soluble CD14 as a Potential Biomarker for Methotrexate Response in Rheumatoid Arthritis. *Front Immunol* (2021) 12:1–15. doi: 10.3389/fimmu.2021.776879

44. Haskó G, Cronstein B, Filep JG, Ludwig A, Taub DD. Regulation of inflammation by adenosine. (2013) doi: 10.3389/fimmu.2013.00085

45. Ma Y, Wang J, Gao J, Yang H, Wang Y, Manithody C, Li J, Rezaie AR. Antithrombin up-regulates AMP-activated protein kinase signalling during myocardial ischaemia/reperfusion injury. *Thromb Haemost* (2015) 113:338–349. doi: 10.1160/TH14-04-0360

46. Wang J, Wang Y, Wang J, Gao J, Tong C, Manithody C, Li J, Rezaie AR. Antithrombin is protective against myocardial ischemia and reperfusion injury. *J Thromb Haemost* (2013) 11:1020–1028. doi: 10.1111/jth.12243.Antithrombin

47. Mihaylova MM, Shaw RJ. The AMP-activated protein kinase (AMPK) signaling pathway coordinates cell growth, autophagy, & metabolism. *Nat Cell Biol* (2011) 13:1016–1023. doi: 10.1038/ncb2329.The

48. Totan M, Dagdemir A, Ak AR, Albayrak D, Kucukoduk S. Effects of High-Dose Methotrexate on the Hemostatic System in Childhood Acute Lymphoblastic Leukemia. *Med Pediatr Oncol* (2001) 36:429–433. doi: 10.1002/mpo.1106

49. Martinez-Bravo MJ, Wahlund CJE, Qazi KR, Moulder R, Lukic A, Rådmark O, Lahesmaa R, Grunewald J, Eklund A, Gabrielsson S. Pulmonary sarcoidosis is associated with exosomal vitamin D–binding protein and inflammatory molecules. *J Allergy Clin Immunol* (2017) 139:1186–1194. doi: 10.1016/j.jaci.2016.05.051

**Supplemental materials**

Isolation of extracellular vesicle serum fractions

LDL and HDL fractions can be obtained from serum by a DS and MnCl2 solution of DS: 0.05%, MnCl2: 0.05M and DS: 0.65%, MnCl2: 0.2M, respectively. For LDL fraction isolation, 25uL serum was diluted in 95μl phosphate buffered saline (PBS) (Gibco), followed by addition of 5μL magnetic beads (Nanomag®-D plain, 130mm (1:25) (Micromod)). DS and MnCl2 were added into the total volume of 125 μL and were mixed. The mixture was incubated 5 min at room temperature (RT). Subsequently, the samples were placed on a bio-plex handheld magnet (Bio-Rad) and incubated 15 min at RT. The formed pellet is LDL fraction. For HDL isolation, the protocol is repeated when using 115μL supernatant above the LDL pellet. The pellets were lysed with 125μL Roche complete lysis-M with protease inhibitors (Roche). To remove magnetic beads and other debris, samples were centrifugated at 3200xg, 10 min.

Table S1. **Baseline characteristics of discovery cohort of sarcoidosis patients with pulmonary treatment indication and healthy controls**.

| **Parameter** | Patients  (n=32) | Healthy control  (n=47) | p-value |
| --- | --- | --- | --- |
| **Age^a^ (years)** | 45.4 ± 10.5 | 39.2 ± 9.0 | 0.007 |
| **Male sex** | 20 (62.5) | 21 (44.7) | 0.122 |
| **Ever smoker** | 29 (34.5) | 18 (38.3) | 0.739 |
| **Caucasian** | 32 (100.0) | 47 (100.0) | 1.000 |

Data is shown as whole numbers and percentages between brackets. Age is shown as mean ± SD. ^a^Age at time of blood withdrawal


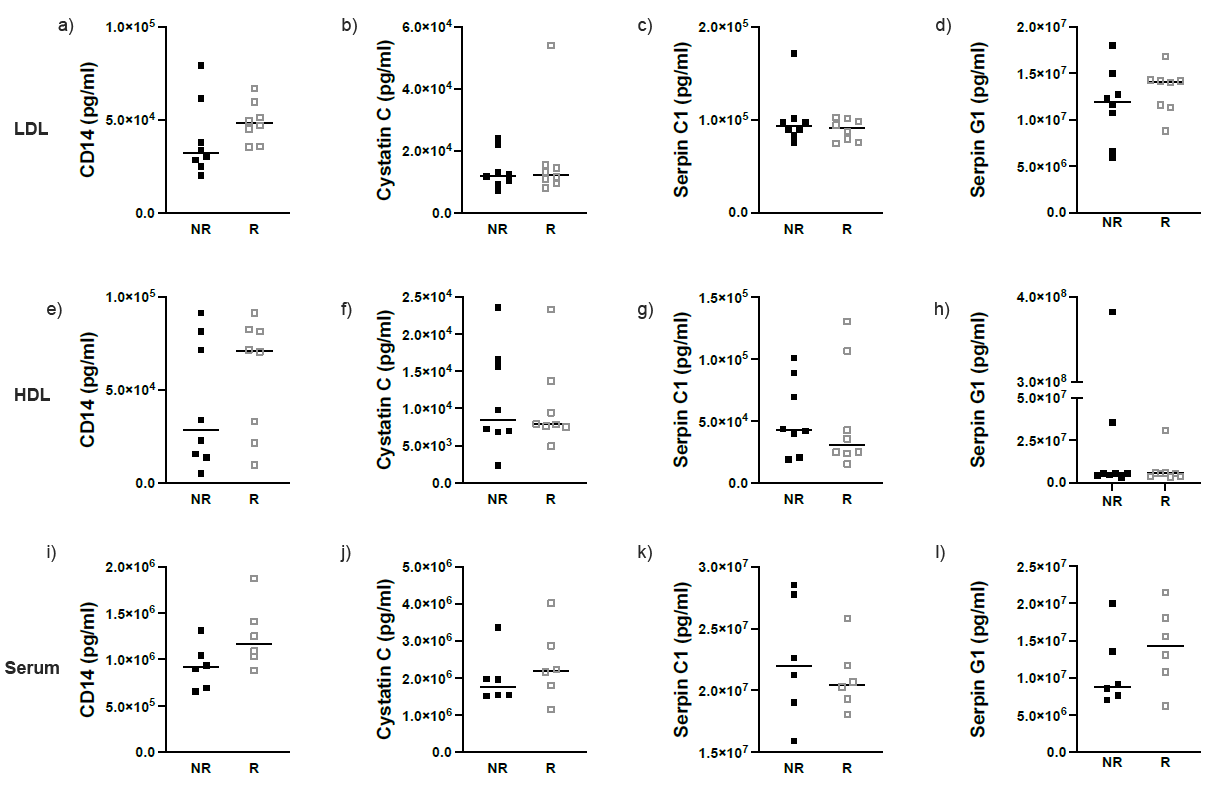


Figure S1. Concentrations of EV biomarkers in patients with sarcoidosis treated with prednisone of the discovery cohort (n=16). Figures a-d) represent protein levels measured in the LDL fraction, figures e-h) represent protein levels measured in the HDL sub fraction and figures i-l) represent protein concentrations measured in whole serum. NR = non-responder, R= responder


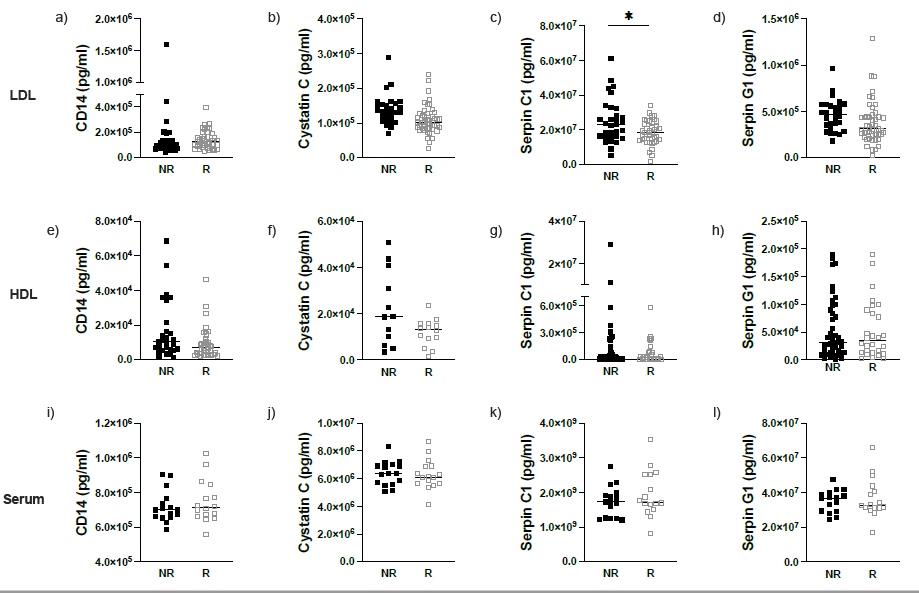


Figure S2. Concentrations of EV biomarkers in patients with pulmonary sarcoidosis treated with MTX of the replication cohort. Figures a-d) represent protein levels measured in the LDL fraction, figures e-h) represent protein levels measured in the HDL sub fraction, and figures i-l) represent protein levels measured in whole serum. NR = non-responder, R = responder. *p<0.05
