# Supplementary figures and images for: Decreased serpin C1 in extracellular vesicles predicts response to methotrexate treatment in patients with pulmonary sarcoidosis
Source: Respir Res. 2024 Apr 16;25:166. doi: 10.1186/s12931-024-02809-y (PMC11020913; doi:10.1186/s12931-024-02809-y)

LDL

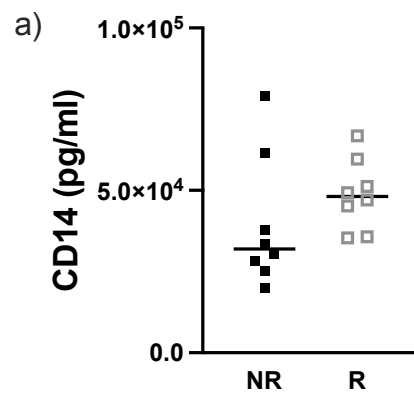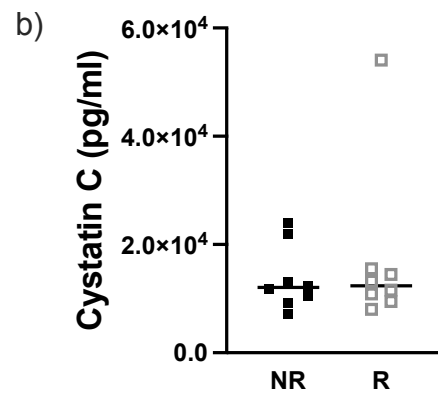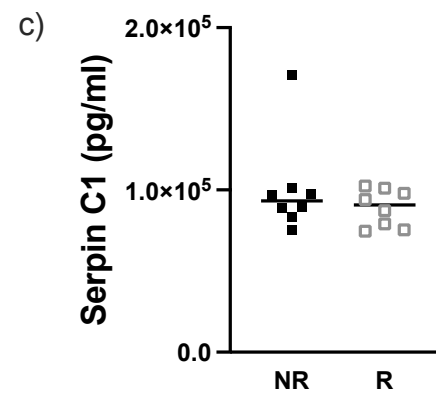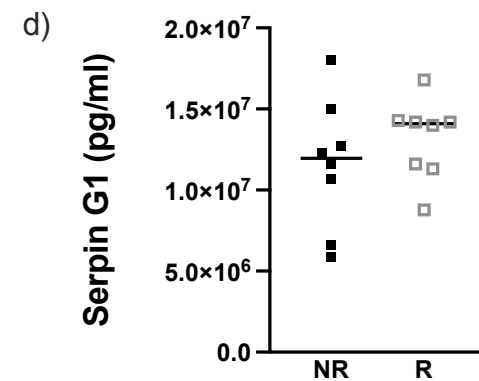

HDL

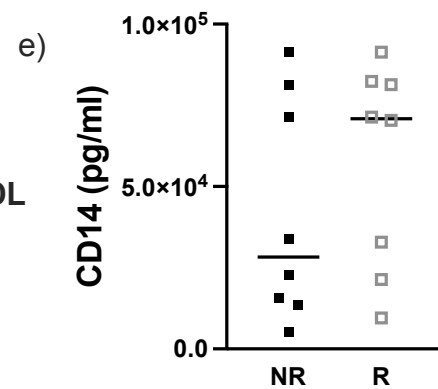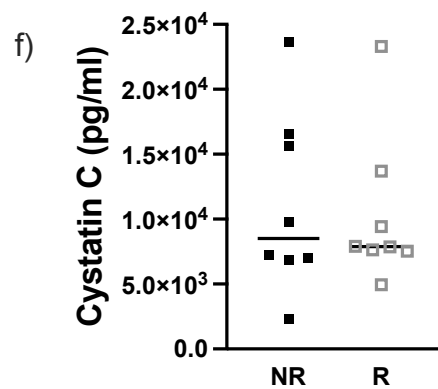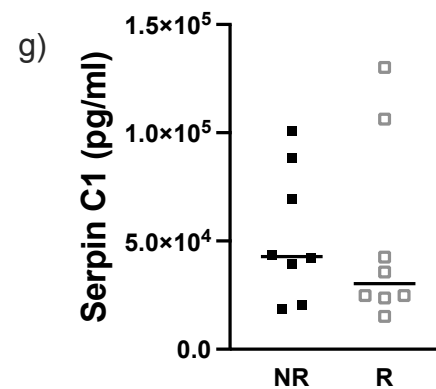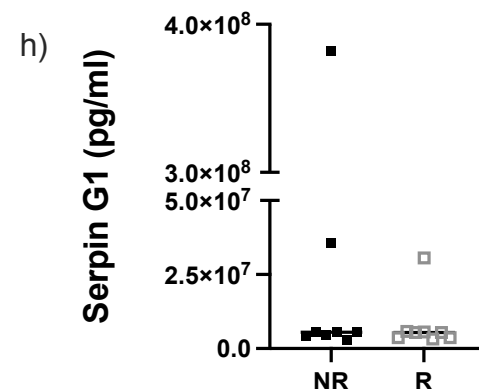

Serum

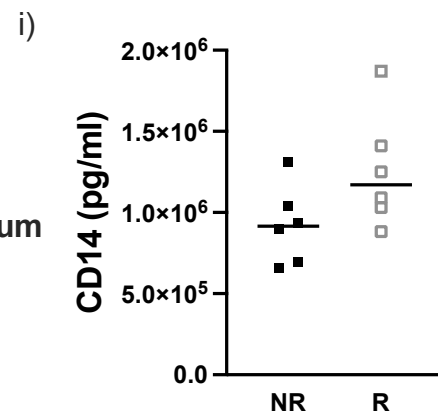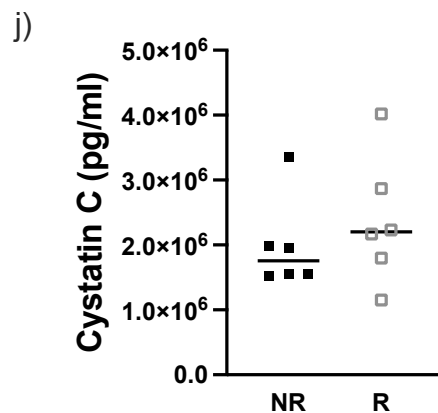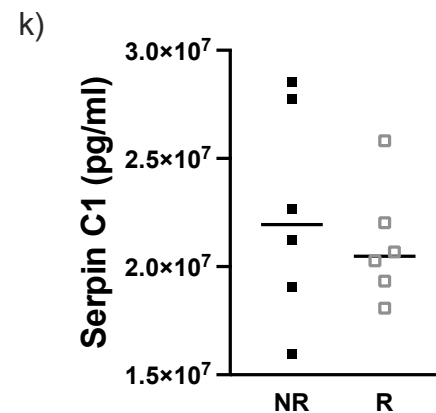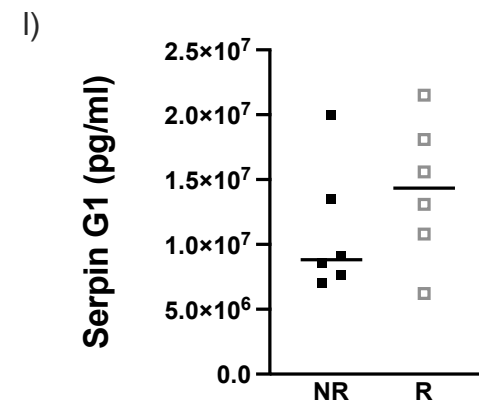

Supplement: Supplementary file 1 — Additional file 1: Isolation of extracellular vesicle serum fractions. LDL and HDL fractions can be obtained from serum by a DS and MnCl2 solution of DS: 0.05%, MnCl2: 0.05 M and DS: 0.65%, MnCl2: 0.2 M, respectively. For LDL fraction isolation, 25uL serum was diluted in 95 μl phosphate buffered saline (PBS) (Gibco), followed by addition of 5 μL magnetic beads (Nanomag®-D plain, 130 mm (1:25) (Micromod)). DS and MnCl2 were added into the total volume of 125 μL and were mixed. The mixture was incubated 5 min at room temperature (RT). Subsequently, the samples were placed on a bio-plex handheld magnet (Bio-Rad) and incubated 15 min at RT. The formed pellet is LDL fraction. For HDL isolation, the protocol is repeated when using 115 μL supernatant above the LDL pellet. The pellets were lysed with 125 μL Roche complete lysis-M with protease inhibitors (Roche). To remove magnetic beads and other debris, samples were centrifugated at 3200×g, 10 min. Table S1. Baseline characteristics of discovery cohort of sarcoidosis patients with pulmonary treatment indication and healthy controls. Fig. S1. Concentrations of EV biomarkers in patients with sarcoidosis treated with prednisone of the discovery cohort (n = 16). Figures a-d) represent protein levels measured in the LDL fraction, figures e-h) represent protein levels measured in the HDL sub fraction and figures i-l) represent protein concentrations measured in whole serum. NR = non-responder, R = responder. Fig. S2. Concentrations of EV biomarkers in patients with pulmonary sarcoidosis treated with MTX of the replication cohort. Figures a-d) represent protein levels measured in the LDL fraction, figures e-h) represent protein levels measured in the HDL sub fraction, and figures i-l) represent protein levels measured in whole serum. NR = non-responder, R = responder. *p < 0.05. [file 12931_2024_2809_MOESM1_ESM.zip › Supl. figure S1. disc. cohort PRED.pdf]

LDL

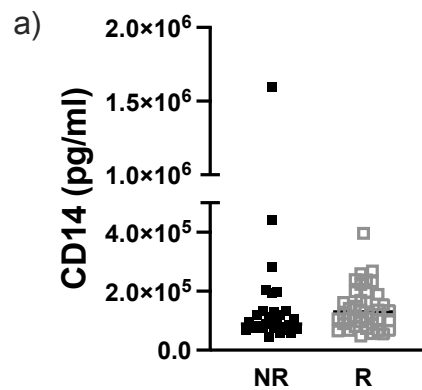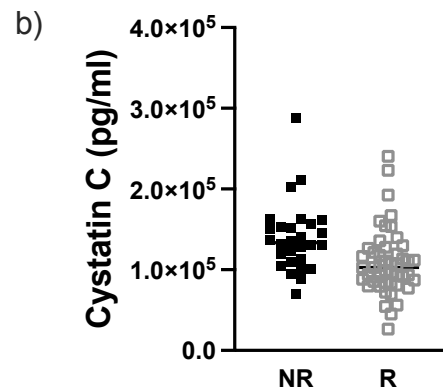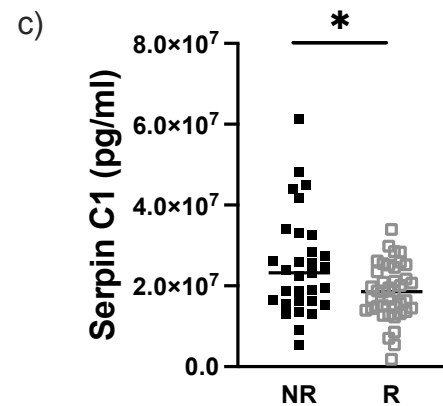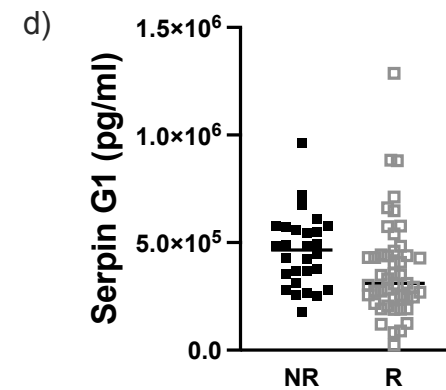

HDL

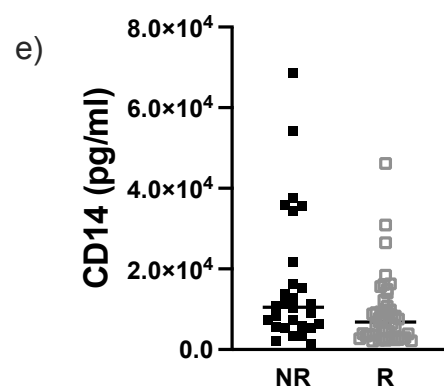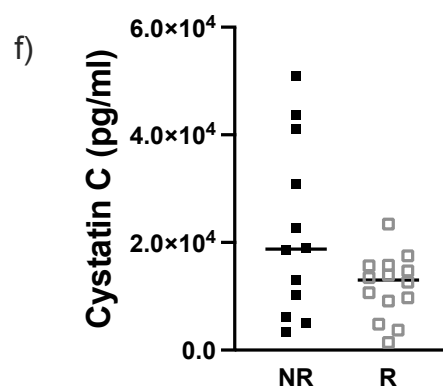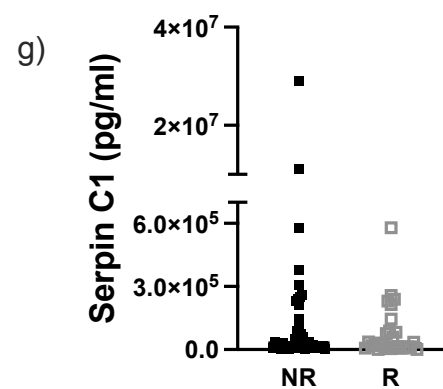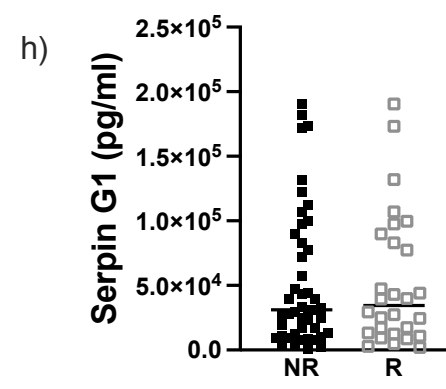

Serum

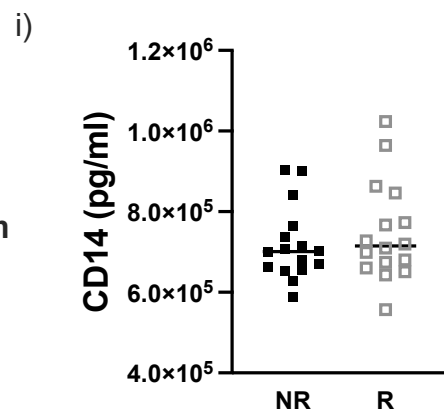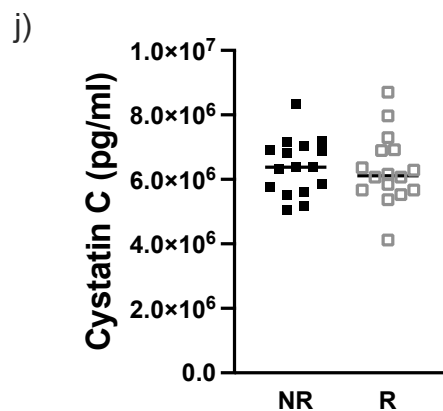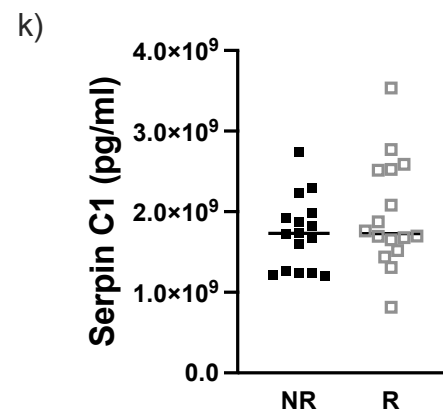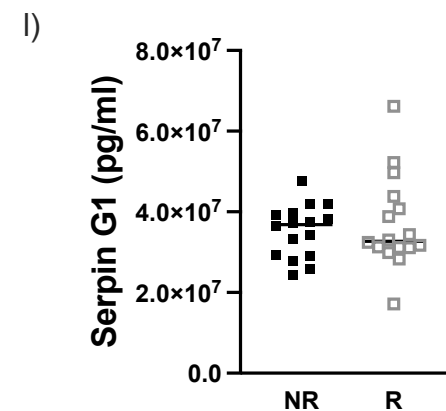

Supplement: Supplementary file 1 — Additional file 1: Isolation of extracellular vesicle serum fractions. LDL and HDL fractions can be obtained from serum by a DS and MnCl2 solution of DS: 0.05%, MnCl2: 0.05 M and DS: 0.65%, MnCl2: 0.2 M, respectively. For LDL fraction isolation, 25uL serum was diluted in 95 μl phosphate buffered saline (PBS) (Gibco), followed by addition of 5 μL magnetic beads (Nanomag®-D plain, 130 mm (1:25) (Micromod)). DS and MnCl2 were added into the total volume of 125 μL and were mixed. The mixture was incubated 5 min at room temperature (RT). Subsequently, the samples were placed on a bio-plex handheld magnet (Bio-Rad) and incubated 15 min at RT. The formed pellet is LDL fraction. For HDL isolation, the protocol is repeated when using 115 μL supernatant above the LDL pellet. The pellets were lysed with 125 μL Roche complete lysis-M with protease inhibitors (Roche). To remove magnetic beads and other debris, samples were centrifugated at 3200×g, 10 min. Table S1. Baseline characteristics of discovery cohort of sarcoidosis patients with pulmonary treatment indication and healthy controls. Fig. S1. Concentrations of EV biomarkers in patients with sarcoidosis treated with prednisone of the discovery cohort (n = 16). Figures a-d) represent protein levels measured in the LDL fraction, figures e-h) represent protein levels measured in the HDL sub fraction and figures i-l) represent protein concentrations measured in whole serum. NR = non-responder, R = responder. Fig. S2. Concentrations of EV biomarkers in patients with pulmonary sarcoidosis treated with MTX of the replication cohort. Figures a-d) represent protein levels measured in the LDL fraction, figures e-h) represent protein levels measured in the HDL sub fraction, and figures i-l) represent protein levels measured in whole serum. NR = non-responder, R = responder. *p < 0.05. [file 12931_2024_2809_MOESM1_ESM.zip › Supl. figure S2 repl. cohort.pdf]
